# Supplementary figures and images for: Biodiversity assessment and environmental risk analysis of the single line transgenic pod borer resistant cowpea
Source: PeerJ. 2024 Oct 18;12:e18094. doi: 10.7717/peerj.18094 (PMC11493023; doi:10.7717/peerj.18094)

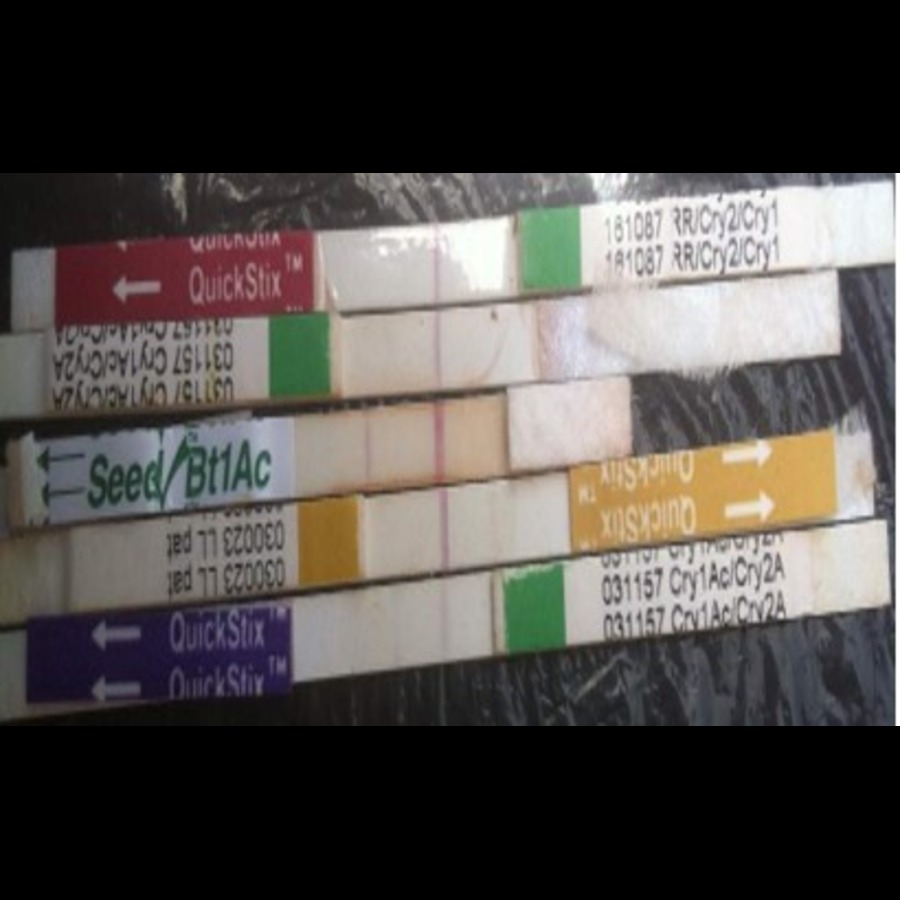

Supplement: Supplemental Information 1 [file peerj-12-18094-s001.jpg]

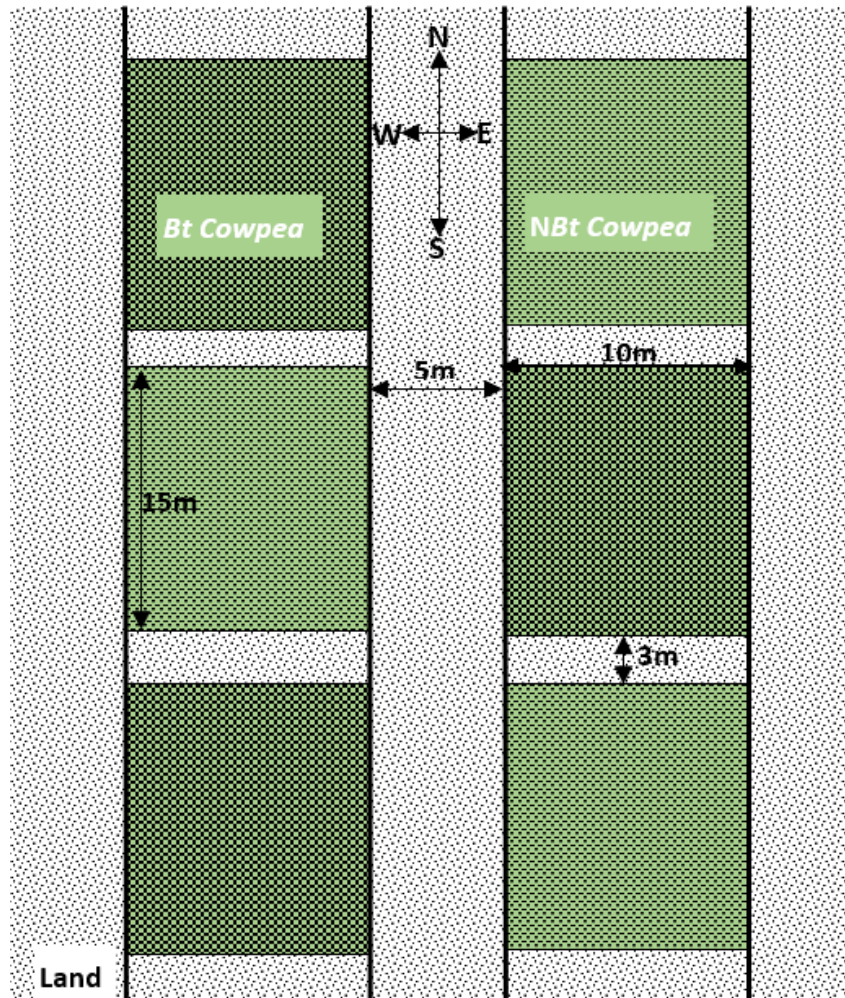

Supplement: Supplemental Information 2 [file peerj-12-18094-s002.pdf]
