# Supplementary material for: Biodiversity assessment and environmental risk analysis of the single line transgenic pod borer resistant cowpea
Source: PeerJ. 2024 Oct 18;12:e18094. doi: 10.7717/peerj.18094 (PMC11493023; doi:10.7717/peerj.18094)
Supplement: Supplemental Information 3 [file peerj-12-18094-s003.docx]

**Insect Capturing Trend March to June 2023**

|  |  |  |  |  |  |  |  |  |  |  |  |  |  |  |  |  |  |  |  |  |  |  |  |  |  |  |  |  |  |
| --- | --- | --- | --- | --- | --- | --- | --- | --- | --- | --- | --- | --- | --- | --- | --- | --- | --- | --- | --- | --- | --- | --- | --- | --- | --- | --- | --- | --- | --- |
| WK | Farm |  |  |  |  |  |  |  |  |  |  |  |  |  |  |  |  |  |  |  |  |  |  |  |  |  |  |  |  |
|  |  |  | **1** | **2** | **3** | **4** | **5** | **6** | **7** | **8** | **9** | **10** | **11** | **12** | **13** | **14** | **15** | **16** | **17** | **18** | **19** | **20** | **21** | **22** | **23** | **24** | **25** | **26** | **27** |
|  |  |  | ***PP*** | **GS** | **GB** | **CS** | **SaC** | **AE** | **ZV** | **EL** | **DA** | **MD** | **AC** | **AD** | **MB** | **SS** | **OT** | **DC** | **JO** | **BT** | **CM** | **HE** | **CC** | **SL** | **CB** | **CaM** | **CaC** | **LM** | **vf** |
| **1**  25  Mar | **1** | *Bt* | **4** | **2** | **6** | **0** | **23** | **00** | **3** | **2** | **1** | **10** | **11** | **4** | **15** | **0** | **5** | **4** | **0** | **0** | **0** | **0** | **0** | **0** | **0** | **0** | **49** | **0** | **0** |
|  |  | NBt | **3** | **1** | **4** | **0** | **24** | **1** | **2** | **1** | **1** | **12** | **8** | **4** | **9** | **0** | **3** | **3** | **0** | **0** | **0** | **0** | **0** | **0** | **1** | **0** | **48** | **0** | **0** |
|  | **2** | Bt | **4** | **2** | **7** | **2** | **20** | **0** | **5** | **1** | **0** | **13** | **9** | **5** | **11** | **0** | **5** | **1** | **2** | **0** | **0** | **0** | **0** | **1** | **0** | **0** | **44** | **0** | **0** |
|  |  | NBt | **2** | **3** | **5** | **3** | **22** | **0** | **2** | **3** | **0** | **9** | **6** | **1** | **10** | **0** | **1** | **1** | **0** | **0** | **0** | **0** | **0** | **1** | **0** | **0** | **37** | **0** | **0** |
|  | **3** | Bt | **3** | **2** | **5** | **0** | **20** | **1** | **4** | **2** | **1** | **8** | **8** | **4** | **8** | **0** | **3** | **0** | **0** | **0** | **0** | **0** | **0** | **2** | **0** | **2** | **20** | **0** | **0** |
|  |  | NBt | **1** | **2** | **6** | **1** | **19** | **0** | **1** | **0** | **0** | **7** | **7** | **2** | **9** | **0** | **4** | **0** | **1** | **0** | **0** | **0** | **0** | **0** | **0** | **1** | **23** | **0** | **0** |
| **2**  1  Apr | **1** | Bt | **4** | **3** | **7** | **2** | **24** | **1** | **3** | **1** | **3** | **11** | **11** | **3** | **16** | **0** | **4** | **4** | **2** | **0** | **0** | **1** | **0** | **1** | **1** | **0** | **53** | **0** | **0** |
|  |  | NBt | **3** | **2** | **3** | **3** | **21** | **2** | **2** | **4** | **2** | **13** | **10** | **2** | **12** | **0** | **3** | **3** | **1** | **0** | **0** | **0** | **1** | **0** | **1** | **0** | **50** | **0** | **0** |
|  | **2** | Bt | **2** | **4** |  | **2** | **22** | **1** | **2** | **4** | **2** | **11** | **8** | **6** | **11** | **0** | **4** | **0** | **2** | **0** | **0** | **0** | **2** | **1** | **1** | **0** | **41** | **0** | **0** |
|  |  | NBt | **3** | **2** | **1** | **1** | **19** | **2** | **3** | **2** | **1** | **12** | **10** | **3** | **9** | **0** | **2** | **1** | **1** | **0** | **0** | **0** | **0** | **1** | **0** | **0** | **39** | **0** | **0** |
|  | **3** | Bt | **5** | **3** | **5** | **2** | **22** | **1** | **3** | **3** | **0** | **10** | **8** | **2** | **10** | **0** | **5** | **1** | **1** | **0** | **0** | **1** | **1** | **1** | **0** | **1** | **23** | **0** | **0** |
|  |  | NBt | **2** | **1** | **4** | **1** | **21** | **0** | **3** | **1** | **1** | **9** | **9** | **3** | **10** | **0** | **2** | **0** | **1** | **0** | **0** | **1** | **0** | **0** | **1** | **1** | **20** | **0** | **0** |
| **3**  8  Apr | **1** | *Bt* | **4** | **3** | **6** | **2** | **24** | **2** | **2** | **2** | **4** | **12** | **12** | **5** | **15** | **0** | **6** | **6** | **3** | **0** | **0** | **2** | **2** | **0** | **2** | **0** | **50** | **0** | **0** |
|  |  | NBt | **5** | **2** | **5** | **1** | **24** | **1** | **2** | **3** | **3** | **14** | **10** | **1** | **13** | **0** | **4** | **5** | **2** | **0** | **0** | **1** | **1** | **1** | **1** | **0** | **51** | **0** | **0** |
|  | **2** | *Bt* | **5** | **4** | **6** | **3** | **23** | **2** | **4** | **6** | **2** | **10** | **12** | **3** | **11** | **0** | **8** | **2** | **4** | **0** | **0** | **0** | **0** | **2** | **0** | **0** | **44** | **0** | **0** |
|  |  | NBt | **4** | **3** | **5** | **2** | **19** | **1** | **3** | **5** | **1** | **9** | **9** | **5** | **11** | **0** | **6** | **2** | **2** | **0** | **0** | **0** | **1** | **1** | **1** | **0** | **41** | **0** | **0** |
|  | **3** | Bt | **5** | **2** | **7** | **1** | **22** | **0** | **4** | **3** | **3** | **13** | **10** | **4** | **13** | **0** | **6** | **1** | **1** | **1** | **0** | **1** | **0** | **0** | **2** | **1** | **22** | **0** | **0** |
|  |  | NBt | **4** | **1** | **6** | **0** | **20** | **2** | **2** | **2** | **1** | **9** | **10** | **2** | **9** | **0** | **5** | **1** | **3** |  | **0** | **0** | **1** | **1** | **1** | **0** | **19** | **0** | **0** |
| **4**  15  Apr | **1** | Bt | **12** | **06** | **12** | **3** | **26** | **4** | **7** | **6** | **6** | **17** | **15** | **8** | **18** | **2** | **8** | **8** | **6** | **2** | **3** | **4** | **3** | **2** | **3** | **1** | **55** | **1** | **0** |
|  |  | NBt | **7** | **4** | **6** | **2** | **22** | **1** | **2** | **2** | **3** | **11** | **9** | **4** | **11** | **0** | **5** | **4** | **2** | **0** | **0** | **1** | **1** | **0** | **1** | **0** | **50** | **0** | **0** |
|  | **2** | Bt | **10** | **07** | **11** | **4** | **26** | **3** | **9** | **4** | **7** | **15** | **15** | **6** | **15** | **2** | **10** | **5** | **4** | **2** | **4** | **4** | **4** | **5** | **5** | **2** | **47** | **2** | **0** |
|  |  | NBt | **07** | **05** | **8** | **4** | **19** | **1** | **5** | **5** | **2** | **11** | **11** | **4** | **9** | **1** | **7** | **1** | **1** | **2** | **2** | **2** | **1** | **1** | **2** | **0** | **43** | **0** | **0** |
|  | **3** | Bt | **12** | **05** | **10** | **6** | **27** | **5** | **7** | **7** | **4** | **16** | **12** | **6** | **15** | **1** | **10** | **6** | **5** | **1** | **1** | **1** | **2** | **5** | **1** | **2** | **57** | **2** | **0** |
|  |  | NBt | **10** | **3** | **07** | **3** | **20** | **1** | **4** | **3** | **1** | **10** | **10** | **3** | **9** | **0** | **6** | **3** | **2** | **0** | **0** | **0** | **1** | **2** | **0** | **1** | **19** | **0** | **0** |
| **5**  22  Apr | **1** | **Bt** | **15** | **09** | **15** | **7** | **28** | **7** | **9** | **11** | **12** | **20** | **17** | **10** | **22** | **2** | **13** | **11** | **8** | **4** | **3** | **4** | **6** | **5** | **5** | **3** | **60** | **3** | **1** |
|  |  | **NBt** | **10** | **04** | **09** | **2** | **4** | **3** | **3** | **4** | **3** | **11** | **8** | **1** | **13** | **1** | **5** | **3** | **4** | **0** | **1** | **2** | **1** | **1** | **1** | **0** | **48** | **0** | **1** |
|  | **2** | **Bt** | **14** | **08** | **13** | **9** | **30** | **7** | **11** | **9** | **9** | **17** | **18** | **8** | **19** | **4** | **12** | **5** | **6** | **4** | **7** | **5** | **3** | **5** | **7** | **1** | **55** | **4** | **3** |
|  |  | **NBt** | **7** | **6** | **08** | **2** | **19** | **0** | **1** | **2** | **1** | **5** | **9** | **4** | **10** | **0** | **6** | **4** | **2** | **1** | **0** | **1** | **0** | **0** | **1** | **1** | **37** | **0** | **0** |
|  | **3** | **Bt** | **12** | **08** | **14** | **9** | **31** | **8** | **8** | **10** | **4** | **17** | **15** | **9** | **18** | **3** | **13** | **12** | **11** | **6** | **3** | **8** | **6** | **5** | **8** | **5** | **57** | **5** | **0** |
|  |  | **NBt** | **4** | **06** | **11** | **1** | **23** | **2** | **4** | **5** | **3** | **9** | **11** | **5** | **12** |  | **6** | **3** | **3** | **1** | **0** | **1** | **2** | **1** | **2** | **2** | **20** | **0** | **0** |
| **6**  29  Apr | **1** | **Bt** | **12** | **13** | **16** | **9** | **27** | **9** | **8** | **11** | **11** | **20** | **19** |  | **23** | **5** | **10** | **9** | **11** | **4** | **4** | **7** | **7** | **8** | **6** | **3** | **66** | **6** | **3** |
|  |  | **NBt** | **8** | **09** | **10** |  | **9** | **6** | **4** | **6** | **6** | **14** | **13** | **7** | **12** | **2** | **5** | **5** | **4** | **2** | **2** | **2** | **3** | **2** | **3** | **1** | **53** | **1** | **1** |
|  | **2** | **Bt** | **12** | **12** | **15** | **11** | **30** | **8** | **9** | **13** | **12** | **22** | **22** | **10** | **19** | **7** | **12** | **8** | **7** | **5** | **7** | **9** | **8** | **6** | **8** | **4** | **57** | **4** | **3** |
|  |  | **NBt** | **10** | **07** | **10** | **4** | **20** | **2** | **4** | **4** | **4** | **7** | **12** | **3** | **8** | **1** | **3** | **3** | **1** | **1** | **2** | **2** | **1** | **1** | **1** | **1** | **39** | **1** | **1** |
|  | **3** | **Bt** | **12** | **11** | **16** | **12** | **29** | **7** | **9** | **9** | **6** | **20** | **18** | **11** | **20** | **5** | **14** | **13** | **11** | **8** | **5** | **11** | **8** | **7** | **11** | **9** | **59** | **5** | **3** |
|  |  | **N*Bt*** | **11** | **08** | **11** | **4** | **26** | **3** | **2** | **3** | **4** | **13** | **9** | **5** | **14** | **1** | **7** | **5** | **1** | **1** | **1** | **1** | **2** | **2** | **3** | **1** | **25** | **1** | **1** |
| **7**  06  May | **1** | ***Bt*** | **19** | **13** | **18** | **10** | **30** | **9** | **9** | **12** | **15** | **22** | **23** | **12** | **24** | **5** | **12** | **13** | **12** | **8** | **4** | **6** | **9** | **9** | **9** | **7** | **67** | **6** | **2** |
|  |  | **N*Bt*** | **13** | **10** | **12** | **8** | **11** | **5** | **6** | **9** | **5** | **17** | **14** | **6** | **14** | **3** | **5** | **9** | **7** | **3** | **4** | **3** | **3** | **1** | **5** | **3** | **53** | **4** | **3** |
|  | **2** | ***Bt*** | **14** | **14** | **15** | **11** | **32** | **11** | **12** | **14** | **14** | **23** | **24** | **13** | **20** | **9** | **15** | **10** | **7** | **8** | **10** | **11** | **8** | **7** | **9** | **8** | **56** | **7** | **4** |
|  |  | **N*Bt*** | **6** | **08** | **12** | **5** | **24** | **3** | **5** | **7** | **3** | **8** | **13** | **5** | **11** | **3** | **5** | **5** | **2** | **3** | **2** | **2** | **4** | **3** | **2** | **4** | **43** | **2** | **2** |
|  | **3** | ***Bt*** | **12** | **12** | **18** | **12** | **33** | **14** | **13** | **9** | **7** | **24** | **15** | **13** | **23** | **3** | **14** | **15** | **16** | **10** | **7** | **13** | **8** | **10** | **13** | **10** | **62** | **8** | **4** |
|  |  | **N*Bt*** | **09** | **10** | **13** | **4** | **28** | **5** | **3** | **5** | **8** | **15** | **11** | **7** | **15** | **1** | **8** | **5** | **4** | **3** | **3** | **3** | **5** | **2** | **4** | **2** | **31** | **3** | **1** |
| **8**  13  May | **1** | ***Bt*** | **16** | **13** | **17** | **10** | **32** | **10** | **11** | **11** | **17** | **20** | **25** | **13** | **25** | **8** | **12** | **16** | **13** | **10** | **8** | **8** | **11** | **10** | **5** | **9** | **70** | **7** | **3** |
|  |  | **N*Bt*** | **11** | **11** | **11** | **6** | **13** | **11** | **09** | **12** | **15** | **17** | **14** | **11** | **14** | **7** | **11** | **16** | **07** | **06** | **05** | **07** | **08** | **06** | **07** | **07** | **59** | **5** | **6** |
|  | **2** | ***Bt*** | **12** | **18** | **16** | **10** | **16** | **14** | **14** | **16** | **17** | **21** | **23** | **13** | **23** | **09** | **14** | **13** | **10** | **10** | **08** | **13** | **14** | **10** | **10** | **11** | **50** | **3** | **5** |
|  |  | **NBt** | **8** | **15** | **08** | **7** | **22** | **8** | **09** | **15** | **08** | **17** | **15** | **08** | **11** | **05** | **09** | **10** | **09** | **08** | **06** | **08** | **09** | **07** | **10** | **07** | **49** | **6** | **4** |
|  | **3** | **Bt** | **15** | **12** | **16** | **16** | **34** | **18** | **14** | **13** | **19** | **23** | **21** | **13** | **22** | **09** | **17** | **17** | **17** | **12** | **11** | **15** | **12** | **11** | **15** | **10** | **60** | **12** | **7** |
|  |  | **NBt** | **07** | **08** | **11** | **08** | **25** | **12** | **07** | **8** | **12** | **14** | **12** | **10** | **18** | **07** | **10** | **15** | **15** | **08** | **09** | **08** | **10** | **10** | **08** | **10** | **43** | **08** | **5** |
| **9**  20  May | **1** | ***Bt*** | **22** | **17** | **15** | **12** | **35** | **13** | **16** | **13** | **21** | **24** | **22** | **17** | **25** | **12** | **15** | **16** | **17** | **13** | **10** | **11** | **11** | **12** | **12** | **10** | **72** | **12** | **5** |
|  |  | **N*Bt*** | **12** | **13** | **11** | **10** | **12** | **12** | **11** | **10** | **12** | **19** | **13** | **14** | **16** | **05** | **10** | **08** | **13** | **07** | **09** | **09** | **06** | **06** | **06** | **09** | **68** | **10** | **4** |
|  | **2** | ***Bt*** | **13** | **16** | **18** | **14** | **32** | **15** | **13** | **16** | **18** | **21** | **24** | **13** | **21** | **11** | **19** | **15** | **14** | **14** | **12** | **16** | **16** | **12** | **14** | **12** | **58** | **10** | **4** |
|  |  | **NBt** | **07** | **09** | **10** | **09** | **23** | **08** | **10** | **08** | **14** | **15** | **11** | **08** | **18** | **10** | **17** | **13** | **13** | **11** | **10** | **08** | **12** | **10** | **12** | **06** | **46** | **07** | **5** |
|  | **3** | **Bt** | **19** | **18** | **20** | **18** | **34** | **23** | **14** | **12** | **14** | **26** | **25** | **17** | **27** | **08** | **20** | **17** | **17** | **13** | **11** | **18** | **14** | **13** | **16** | **15** | **62** | **10** | **6** |
|  |  | **NBt** | **12** | **14** | **17** | **15** | **25** | **14** | **12** | **08** | **13** | **17** | **17** | **10** | **20** | **07** | **10** | **15** | **08** | **08** | **09** | **1** | **12** | **10** | **10** | **13** | **49** | **10** | **3** |
| **10**  27  May | **1** | **Bt** | **21** | **17** | **22** | **16** | **37** | **17** | **15** | **14** | **18** | **24** | **30** | **15** | **23** | **13** | **18** | **18** | **16** | **17** | **12** | **14** | **18** | **14** | **14** | **11** | **72** | **12** |  |
|  |  | **NBt** | **12** | **11** | **10** | **10** | **11** | **14** | **14** | **10** | **13** | **21** | **28** | **13** | **13** | **10** | **07** | **14** | **14** | **14** | **10** | **11** | **17** | **12** | **10** | **08** | **69** | **08** | **1** |
|  | **2** | **Bt** | **12** | **19** | **20** | **15** | **36** | **14** | **12** | **20** | **21** | **19** | **31** | **17** | **24** | **12** | **22** | **16** | **16** | **15** | **13** | **16** | **19** | **16** | **16** | **15** | **68** | **07** | **3** |
|  |  | **NBt** | **07** | **12** | **12** | **10** | **23** | **10** | **10** | **20** | **14** | **11** | **17** | **10** | **14** | **11** | **20** | **14** | **13** | **12** | **11** | **11** | **12** | **11** | **10** | **11** | **56** | **08** | **4** |
|  | **3** | **Bt** | **19** | **22** | **24** | **19** | **34** | **22** | **14** | **16** | **22** | **24** | **22** | **18** | **27** | **12** | **22** | **16** | **16** | **15** | **15** | **17** | **15** | **13** | **14** | **15** | **69** | **12** | **7** |
|  |  | **NBt** | **06** | **14** | **19** | **12** | **25** | **16** | **12** | **11** | **15** | **19** | **11** | **10** | **19** | **11** | **18** | **08** | **10** | **09** | **14** | **10** | **11** | **7** | **10** | **10** | **35** | **10** | **6** |
| **11**  03  June | **1** | **Bt** | **22** | **20** | **26** | **20** | **33** | **15** | **23** | **15** | **20** | **27** | **31** | **17** | **24** | **15** | **20** | **14** | **14** | **16** | **13** | **16** | **14** | **17** | **17** | **10** | **73** | **13** | **6** |
|  |  | **NBt** | **14** | **14** | **22** | **16** | **15** | **12** | **14** | **12** | **12** | **26** | **19** | **13** | **18** | **10** | **17** | **09** | **11** | **14** | **11** | **10** | **10** | **10** | **12** | **10** | **70** | **10** | **4** |
|  | **2** | **Bt** | **15** | **16** | **21** | **21** | **37** | **16** | **15** | **21** | **23** | **24** | **32** | **18** | **26** | **14** | **23** | **15** | **17** | **16** | **11** | **19** | **19** | **22** | **18** | **13** | **64** | **08** | **3** |
|  |  | **NBt** | **09** | **10** | **19** | **17** | **21** | **13** | **10** | **11** | **12** | **21** | **19** | **14** | **19** | **12** | **19** | **14** | **13** | **15** | **09** | **10** | **12** | **12** | **09** | **10** | **58** | **05** | **2** |
|  | **3** | **Bt** | **20** | **21** | **19** | **21** | **36** | **21** | **19** | **15** | **25** | **20** | **22** | **20** | **31** | **13** | **20** | **18** | **17** | **13** | **15** | **18** | **16** | **15** | **21** | **22** | **65** | **11** | **6** |
|  |  | **NBt** | **06** | **15** | **15** | **15** | **22** | **13** | **15** | **13** | **11** | **17** | **16** | **15** | **9** | **11** | **16** | **11** | **15** | **07** | **13** | **12** | **15** | **6** | **16** | **17** | **56** | **04** | **7** |
| **12**  10  June | **1** | **Bt** | **20** | **20** | **20** | **21** | **38** | **17** | **21** | **16** | **21** | **31** | **28** | **16** | **25** | **17** | **23** | **21** | **19** | **1** | **17** | **19** | **17** | **18** | **20** | **13** | **76** | **12** | **8** |
|  |  | **NBt** | **20** | **14** | **07** | **14** | **34** | **14** | **18** | **11** | **16** | **28** | **16** | **14** | **11** | **09** | **15** | **20** | **13** | **08** | **15** | **15** | **15** | **13** | **17** | **08** | **73** | **10** | **6** |
|  | **2** | **Bt** | **22** | **19** | **21** | **22** | **39** | **15** | **23** | **23** | **23** | **26** | **32** | **18** | **23** | **16** | **24** | **21** | **17** | **13** | **12** | **20** | **21** | **24** | **22** | **15** | **65** | **10** | **8** |
|  |  | **NBt** | **13** | **12** | **12** | **14** | **233** | **15** | **18** | **12** | **16** | **28** | **18** | **13** | **20** | **15** | **23** | **18** | **13** | **09** | **10** | **15** | **13** | **11** | **19** | **12** | **53** | **08** | **8** |
|  | **3** | **Bt** | **24** | **22** | **19** | **24** | **36** | **23** | **21** | **13** | **24** | **23** | **27** | **20** | **29** | **14** | **20** | **17** | **20** | **12** | **11** | **20** | **16** | **15** | **19** | **22** | **66** | **14** | **9** |
|  |  | **NBt** | **11** | **16** | **13** | **19** | **35** | **20** | **17** | **12** | **17** | **12** | **23** | **11** | **19** | **12** | **19** | **13** | **18** | **13** | **10** | **11** | **13** | **7** | **14** | **16** | **53** | **10** | **6** |
|  |  |  |  |  |  |  |  |  |  |  |  |  |  |  |  |  |  |  |  |  |  |  |  |  |  |  |  |  |  |
|  |  |  |  |  |  |  |  |  |  |  |  |  |  |  |  |  |  |  |  |  |  |  |  |  |  |  |  |  |  |
|  |  |  |  |  |  |  |  |  |  |  |  |  |  |  |  |  |  |  |  |  |  |  |  |  |  |  |  |  |  |
