# Supplementary material for: Biodiversity assessment and environmental risk analysis of the single line transgenic pod borer resistant cowpea
Source: PeerJ. 2024 Oct 18;12:e18094. doi: 10.7717/peerj.18094 (PMC11493023; doi:10.7717/peerj.18094)
Supplement: Supplemental Information 4 [file peerj-12-18094-s004.docx]

**Insect Capturing Trend August to November 2022**

| WK | Farm |  |  |  |  |  |  |  |  |  |  |  |  |  |  |  |  |  |  |  |  |  |  |  |  |  |  |  |  |  |
| --- | --- | --- | --- | --- | --- | --- | --- | --- | --- | --- | --- | --- | --- | --- | --- | --- | --- | --- | --- | --- | --- | --- | --- | --- | --- | --- | --- | --- | --- | --- |
|  |  |  |  |  |  |  |  |  |  |  |  |  |  |  |  |  |  |  |  |  |  |  |  |  |  |  |  |  |  |  |
|  |  |  | **1** | **2** | **3** | **4** | **5** | **6** | **7** | **8** | **9** | **10** | **11** | **12** | **13** | **14** | **15** | **16** | **17** | **18** | **19** | **20** | **21** | **22** | **23** | **24** | **25** | **26** | **27** |  |
|  |  |  | ***PP*** | **GS** | **GB** | **CS** | **SaC** | **AE** | **ZV** | **EL** | **DA** | **MD** | **AC** | **AD** | **MB** | **SS** | **OT** | **DC** | **JO** | **BT** | **CM** | **HE** | **CC** | **SL** | **CB** | **CaM** | **CaC** | **LM** | **vf** |  |
| **1**  25  Mar | **1** | *Bt* | **7** | **7** | **9** | **5** | **24** | **33** | **6** | **7** | **2** | **14** | **17** | **06** | **12** | **1** | **04** | **6** | **0** | **0** | **1** | **0** | **0** | **1** | **2** | **0** | **49** | **1** | **1** |  |
|  |  | NBt | **6** | **5** | **10** | **4** | **21** | **4** | **7** | **6** | **5** | **16** | **14** | **4** | **14** | **1** | **8** | **7** | **0** | **0** | **1** | **0** | **0** | **0** | **1** | **0** | **51** | **1** | **0** |  |
|  | **2** | Bt | **6** | **9** | **8** | **4** | **27** | **3** | **6** | **6** | **4** | **16** | **13** | **8** | **12** | **3** | **6** | **5** | **2** | **1** | **0** | **0** | **5** | **3** | **3** | **1** | **48** | **0** | **0** |  |
|  |  | NBt | **6** | **6** | **11** | **6** | **22** | **2** | **4** | **4** | **6** | **15** | **12** | **6** | **07** | **4** | **7** | **3** | **3** | **2** | **0** | **0** | **2** | **1** | **1** | **0** | **32** | **0** | **2** |  |
|  | **3** | Bt | **5** | **8** | **8** | **5** | **26** | **3** | **6** | **6** | **6** | **12** | **08** | **7** | **14** | **2** | **9** | **1** | **2** | **0** | **1** | **0** | **3** | **0** | **1** | **2** | **20** | **2** | **1** |  |
|  |  | NBt | **3** | **9** | **7** | **5** | **27** | **5** | **7** | **4** | **3** | **09** | **13** | **4** | **11** | **1** | **9** | **1** | **4** | **0** | **0** | **0** | **4** | **4** | **3** | **3** | **26** | **1** | **1** |  |
| **2**  1  Apr | **1** | Bt | **10** | **6** | **9** | **7** | **22** | **2** | **5** | **6** | **7** | **12** | **10** | **7** | **19** | **2** | **9** | **8** | **2** | **2** | **0** | **1** | **2** | **2** | **5** | **0** | **47** | **0** | **0** |  |
|  |  | NBt | **8** | **6** | **8** | **6** | **26** | **0** | **6** | **6** | **5** | **10** | **10** | **8** | **14** | **3** | **8** | **8** | **3** | **1** | **1** | **1** | **1** | **2** | **4** | **0** | **50** | **1** | **1** |  |
|  | **2** | Bt | **7** | **5** | **8** | **2** | **25** | **4** | **2** |  | **5** | **15** | **12** | **9** | **15** | **0** | **8** | **6** | **5** | **0** | **1** | **2** | **2** | **3** | **3** | **2** | **449** | **1** | **0** |  |
|  |  | NBt | **8** | **4** | **6** | **4** | **20** | **5** | **1** | **5** | **6** | **16** | **12** | **7** | **12** | **2** | **8** | **5** | **3** |  | **0** | **2** | **2** | **2** | **4** | **1** | **44** | **1** | **0** |  |
|  | **3** | Bt | **6** | **7** | **4** | **4** | **27** | **1** | **4** | **4** | **3** | **13** | **11** | **3** | **15** | **3** | **8** | **3** | **4** | **1** | **1** | **1** | **3** | **2** | **3** | **1** | **27** | **0** | **1** |  |
|  |  | NBt | **5** | **7** | **6** | **5** | **25** | **4** | **5** | **3** | **3** | **12** | **13** | **5** | **13** | **2** | **7** | **3** | **3** | **0** | **1** | **1** | **2** | **1** | **3** | **1** | **26** | **1** | **0** |  |
| **3**  8  Apr | **1** | *Bt* | **11** | **6** | **9** | **6** | **19** | **6** | **5** | **6** | **8** | **13** | **15** | **6** | **14** | **2** | **4** | **7** | **6** | **2** | **1** | **1** | **4** | **2** | **4** | **2** | **47** | **2** | **1** |  |
|  |  | NBt | **8** | **7** | **3** | **5** | **22** | **5** | **4** | **6** | **4** | **10** | **14** | **6** | **16** | **3** | **5** | **9** | **5** | **1** | **2** | **3** | **4** | **3** | **4** | **1** | **50** | **2** | **0** |  |
|  | **2** | *Bt* | **9** | **8** | **4** | **6** | **27** | **5** | **8** | **6** | **6** | **15** | **11** | **8** | **16** | **2** | **8** | **7** | **4** | **1** | **1** | **4** | **5** | **5** | **3** | **0** | **49** | **3** | **0** |  |
|  |  | NBt | **10** | **5** | **6** | **6** | **21** | **4** | **8** | **8** | **6** | **15** | **13** | **5** | **15** | **1** | **8** | **6** | **5** | **3** | **0** | **3** | **4** | **2** | **4** | **2** | **44** | **2** | **2** |  |
|  | **3** | Bt | **8** | **7** | **8** | **5** | **23** | **5** | **6** | **7** | **5** | **13** | **12** | **7** | **16** | **1** | **8** | **5** | **5** | **1** | **1** | **3** | **3** | **4** | **4** | **1** | **23** | **2** | **1** |  |
|  |  | NBt | **7** | **6** | **7** | **4** | **20** | **5** | **5** | **5** | **6** | **14** | **10** | **8** | **13** | **2** | **10** | **6** | **3** | **2** | **1** | **3** | **4** | **5** | **5** | **2** | **24** | **1** | **1** |  |
| **4**  15  Apr | **1** | Bt | **12** | **12** | **12** | **8** | **30** | **7** | **11** | **9** | **10** | **17** | **18** | **11** | **18** | **2** | **09** | **12** | **8** | **3** | **5** | **6** | **6** | **7** | **6** | **3** | **53** | **4** | **0** |  |
|  |  | NBt | **9** | **9** | **8** | **8** | **28** | **5** | **6** | **7** | **9** | **15** | **13** | **8** | **15** | **2** | **9** | **9** | **6** | **2** | **4** | **5** | **4** | **6** | **4** | **2** | **54** | **2** | **0** |  |
|  | **2** | Bt | **12** | **12** | **13** | **9** | **30** | **6** | **12** | **9** | **10** | **18** | **22** | **10** | **18** | **4** | **12** | **7** | **5** | **5** | **7** | **7** | **6** | **4** | **6** | **4** | **52** | **4** | **0** |  |
|  |  | NBt | **9** | **8** | **9** | **6** | **25** | **4** | **10** | **8** | **8** | **15** | **15** | **8** | **15** | **3** | **10** | **6** | **4** | **4** | **7** | **6** | **4** | **5** | **6** | **3** | **48** | **3** | **2** |  |
|  | **3** | Bt | **16** | **9** | **10** | **6** | **29** | **9** | **10** | **10** | **6** | **19** | **15** | **9** | **16** | **4** | **12** | **10** | **8** | **4** | **3** | **5** | **5** | **7** | **6** | **3** | **59** | **2** | **1** |  |
|  |  | NBt | **9** | **69** | **6** | **6** | **21** | **5** | **7** | **7** | **5** | **17** | **12** | **8** | **13** | **1** | **11** | **09** | **6** | **5** | **2** | **2** | **3** | **6** | **5** | **4** | **44** | **3** | **0** |  |
| **5**  22  Apr | **1** | **Bt** | **17** | **15** | **10** | **10** | **22** | **7** | **12** | **13** | **15** | **25** | **20** | **13** | **24** | **4** | **15** | **15** | **11** | **3** | **6** | **7** | **8** | **8** | **9** | **6** | **68** | **7** | **4** |  |
|  |  | **NBt** | **15** | **13** | **4** | **7** | **19** | **9** | **10** | **10** | **13** | **19** | **17** | **12** | **23** | **4** | **12** | **10** | **10** | **2** | **4** | **5** | **4** | **5** | **6** | **3** | **559** | **4** | **4** |  |
|  | **2** | **Bt** | **16** | **12** | **15** | **11** | **31** | **11** | **15** | **12** | **12** | **219** | **20** | **12** | **23** | **8** | **15** | **7** | **9** | **7** | **9** | **8** | **9** | **6** | **6** | **5** | **54** | **6** | **5** |  |
|  |  | **NBt** | **14** | **11** | **10** | **8** | **8** | **09** | **12** | **11** | **09** | **20** | **15** | **10** | **22** | **6** | **13** | **6** | **8** | **4** | **7** | **6** | **6** | **5** | **7** | **6** | **42** | **5** | **3** |  |
|  | **3** | **Bt** | **17** | **13** | **13** | **12** | **33** | **9** | **11** | **14** | **07** | **21** | **18** | **12** | **17** | **5** | **15** | **15** | **14** | **9** | **7** | **10** | **6** | **9** | **11** | **4** | **62** | **6** | **3** |  |
|  |  | **NBt** | **14** | **12** | **10** | **09** | **32** | **7** | **11** | **13** | **6** | **20** | **19** | **10** | **138** | **3** | **13** | **12** | **13** | **8** | **5** | **7** | **5** | **8** | **09** | **5** | **37** | **5** | **3** |  |
| **6**  29  Apr | **1** | **Bt** | **20** | **17** | **19** | **11** | **30** | **12** | **11** | **14** | **16** | **24** | **21** | **15** | **24** | **8** | **10** | **13** | **14** | **5** | **7** | **10** | **10** | **13** | **08** | **6** | **57** | **4** | **5** |  |
|  |  | **NBt** | **18** | **15** | **18** | **12** | **23** | **11** | **11** | **12** | **14** | **19** | **19** | **13** | **19** | **6** | **11** | **11** | **13** | **5** | **6** | **08** | **09** | **12** | **11** | **7** | **61** | **5** | **4** |  |
|  | **2** | **Bt** | **15** | **14** | **17** | **13** | **31** | **11** | **11** | **12** | **1** | **25** | **25** | **14** | **23** | **10** | **16** | **11** | **10** | **8** | **11** | **12** | **10** | **6** | **12** | **8** | **58** | **6** | **3** |  |
|  |  | **NBt** | **14** | **15** | **15** | **12** | **27** | **8** | **10** | **9** | **14** | **19** | **22** | **13** | **20** | **7** | **13** | **10** | **11** | **9** | **10** | **13** | **08** | **08** | **10** | **7** | **53** | **7** | **3** |  |
|  | **3** | **Bt** | **19** | **14** | **15** | **14** | **34** | **12** | **13** | **11** | **9** | **22** | **19** | **13** | **26** | **5** | **17** | **17** | **16** | **11** | **10** | **14** | **11** | **10** | **10** | **12** | **63** | **9** | **5** |  |
|  |  | **N*Bt*** | **15** | **15** | **14** | **11** | **30** | **11** | **14** | **10** | **10** | **20** | **18** | **12** | **25** | **6** | **13** | **15** | **13** | **10** | **09** | **10** | **10** | **11** | **11** | **12** | **67** | **7** | **6** |  |
| **7**  06  May | **1** | ***Bt*** | **23** | **20** | **22** | **14** | **30** | **12** | **14** | **15** | **18** | **25** | **25** | **17** | **17** | **11** | **16** | **17** | **16** | **12** | **10** | **10** | **13** | **13** | **12** | **10** | **70** | **10** | **07**  **S** |  |
|  |  | **N*Bt*** | **21** | **19** | **20** | **11** | **17** | **10** | **13** | **14** | **10** | **20** | **17** | **10** | **16** | **10** | **10** | **12** | **10** | **10** | **10** | **10** | **10** | **10** | **10** | **10** | **56** | **10** | **10** |  |
|  | **2** | ***Bt*** | **19** | **18** | **18** | **15** | **14** | **15** | **15** | **18** | **19** | **26** | **27** | **16** | **24** | **13** | **18** | **14** | **12** | **12** | **14** | **14** | **14** | **10** | **14** | **10** | **61** | **10** | **7** |  |
|  |  | **N*Bt*** | **17** | **16** | **17** | **13** | **34** | **13** | **14** | **15** | **12** | **12** | **17** | **10** | **15** | **10** | **10** | **10** | **10** | **10** | **10** | **10** | **10** |  | **10** |  | **47** | **10** | **10** |  |
|  | **3** | ***Bt*** | **23** | **16** | **160** | **17** | **33** | **17** | **15** | **13** | **11** | **26** | **20** | **16** | **25** | **11** | **19** | **18** | **18** | **14** | **12** | **16** | **13** | **14** | **16** | **13** | **66** | **12** | **10** |  |
|  |  | **N*Bt*** | **17** | **14** | **18** | **16** | **32** | **15** | **14** | **11** | **12** | **19** | **14** | **14** | **18** | **10** | **12** | **10** | **10** | **10** | **10** | **10** | **10** | **10** | **10** | **10** | **35** | **10** | **6** |  |
| **8**  13  May | **1** | ***Bt*** | **20** | **18** | **18** | **10** | **37** | **14** | **16** | **15** | **21** | **24** | **27** | **17** | **27** | **12** | **17** | **18** | **16** | **14** | **10** | **12** | **14** | **14** | **9** | **10** | **72** | **10** | **4** |  |
|  |  | **N*Bt*** | **21** | **16** | **17** | **10** | **36** | **12** | **1315** | **14** | **10** | **20** | **16** | **12** | **17** | **10** | **12** | **12** | **10** | **10** | **10** | **10** | **10** | **10** | **10** | **10** | **61** | **10** | **10** |  |
|  | **2** | ***Bt*** | **10** | **13** | **15** | **17** | **34** | **16** | **14** |  | **20** | **23** | **28** | **16** | **26** | **11** | **18** | **16** | **12** | **12** | **12** | **16** | **16** | **13** | **11** | **12** | **54** | **7** | **6** |  |
|  |  | **NBt** | **13** | **14** | **12** | **17** | **33** | **14** | **13** | **20** | **12** | **13** | **14** | **13** | **16** | **10** | **10** | **11** | **10** | **10** | **10** | **10** | **10** | **10** | **10** | **10** | **49** | **10** | **6** |  |
|  | **3** | **Bt** | **20** | **18** | **19** | **19** | **30** | **20** | **15** | **16** | **22** | **27** | **23** | **18** | **26** | **11** | **21** | **20** | **20** | **14** | **14** | **18** | **15** | **14** | **17** | **13** | **67** | **11** | **5** |  |
|  |  | **NBt** | **21** | **17** | **15** | **17** | **32** | **17** | **14** | **139** | **12** | **18** | **15** | **13** | **20** | **10** | **14** | **10** | **10** | **10** | **10** | **10** | **10** | **10** | **10** | **10** | **37** | **10** | **4** |  |
| **9**  20  May | **1** | ***Bt*** | **26** | **22** | **21** | **13** | **37** | **16** | **16** | **15** | **21** | **26** | **29** | **20** | **28** | **14** | **18** | **18** | **18** | **15** | **12** | **10** | **15** | **15** | **14** | **13** | **74** | **13** | **10** |  |
|  |  | **N*Bt*** | **24** | **20** | **20** | **12** | **34** | **14** | **15** | **13** | **12** | **21** | **18** | **14** | **16** | **10** | **10** | **13** | **12** | **10** | **10** | **10** | **10** | **10** | **10** | **10** | **60** | **10** | **6** |  |
|  | **2** | ***Bt*** | **15** | **20** | **20** | **18** | **37** | **20** | **13** | **20** | **20** | **23** | **31** | **16** | **26** | **14** | **20** | **16** | **14** | **12** | **14** | **18** | **20** | **16** | **15** | **14** | **58** | **10** | **8** |  |
|  |  | **NBt** | **149** | **18** | **18** | **16** | **37** | **17** | **12** | **11** | **13** | **14** | **16** | **12** | **18** | **11** | **10** | **10** | **10** | **10** | **10** | **10** | **10** | **10** | **10** | **10** | **50** | **10** | **6** |  |
|  | **3** | **Bt** | **26** | **19** | **20** | **21** | **36** | **23** | **17** | **15** | **24** | **28** | **27** | **18** | **29** | **10** | **22** | **20** | **20** | **16** | **14** | **18** | **16** | **10** | **20** | **16** | **64** | **10** | **10** |  |
|  |  | **NBt** | **27** | **21** | **18** | **20** | **35** | **20** | **16** | **12** | **11** | **17** | **18** | **13** | **21** | **11** | **13** | **14** | **12** | **10** | **10** | **10** | **10** | **10** | **10** | **11** | **35** | **10** | **10** |  |
| **10**  27  May | **1** | **Bt** | **26** | **20** | **26** | **19** | **40** | **17** | **18** | **16** | **23** | **28** | **30** | **19** | **29** | **16** | **20** | **18** | **18** | **16** | **14** | **14** | **16** | **18** | **16** | **14** | **75** | **15** | **6** |  |
|  |  | **NBt** | **24** | **18** | **24** | **20** | **38** | **15** | **17** | **10** | **14** | **21** | **19** | **12** | **17** | **10** | **12** | **12** | **14** | **10** | **10** | **10** | **10** | **10** | **10** | **10** | **63** | **10** | **6** |  |
|  | **2** | **Bt** | **13** | **20** | **18** | **12** | **31** | **14** | **15** | **12** | **21** | **22** | **34** | **18** | **27** | **16** | **21** | **17** | **16** | **14** | **14** | **18** | **20** | **16** | **10** | **16** | **64** | **10** |  |  |
|  |  | **NBt** | **14** | **18** | **19** | **14** | **32** | **15** | **12** | **10** | **12** | **14** | **17** | **11** | **18** | **10** | **12** | **10** | **12** | **10** | **12** | **10** | **12** | **12** | **10** | **10** | **53** | **10** | **10** |  |
|  | **3** | **Bt** | **23** | **17** | **20** | **23** | **37** | **25** | **17** | **18** | **25** | **27** | **28** | **21** | **30** | **14** | **24** | **18** | **18** | **18** | **15** | **19** | **18** | **14** | **20** | **18** | **67** | **13** | **9** |  |
|  |  | **NBt** | **20** | **16** | **19** | **23** | **34** | **23** | **15** | **10** | **13** | **18** | **17** | **14** | **20** | **10** | **15** | **15** | **10** | **10** | **10** | **12** | **12** | **10** | **10** | **13** | **40** | **8** | **5** |  |
| **11**  03  June | **1** | **Bt** | **25** | **22** | **23** | **17** | **38** | **18** | **15** | **17** | **22** | **30** | **34** | **17** | **26** | **19** | **22** | **19** | **18** | **16** | **16** | **18** | **18** | **20** | **20** | **15** | **79** | **16** | **10** |  |
|  |  | **NBt** | **26** | **20** | **22** | **17** | **36** | **19** | **17** | **14** | **14** | **20** | **20** | **12** | **16** | **13** | **10** | **14** | **15** | **12** | **10** | **10** | **13** | **12** | **10** | **19** | **66** | **10** | **7** |  |
|  | **2** | **Bt** | **17** | **18** | **25** | **19** | **35** | **20** | **15** | **24** | **25** | **24** | **33** | **21** | **28** | **18** | **22** | **18** | **16** | **16** | **13** | **18** | **21** | **23** | **20** | **14** | **67** | **13** | **7** |  |
|  |  | **NBt** | **16** | **19** | **2** | **17** | **33** | **18** | **14** | **15** | **15** | **17** | **18** | **13** | **22** | **12** | **13** | **12** | **10** | **10** | **10** | **10** | **15** | **14** | **12** | **10** | **52** | **10** | **5** |  |
|  | **3** | **Bt** | **27** | **21** | **22** | **24** | **38** | **25** | **21** | **14** | **27** | **26** | **29** | **22** | **34** | **16** | **24** | **18** | **20** | **16** | **14** | **21** | **20** | **16** | **23** | **21** | **69** | **15** | **10** |  |
|  |  | **NBt** | **21** | **20** | **20** | **26** | **33** | **23** | **18** | **11** | **12** | **17** | **19** | **15** | **19** | **12** | **16** | **14** | **14** | **10** | **10** | **15** | **14** | **10** | **10** | **14** | **43** | **10** | **9** |  |
| **12**  10  June | **1** | **Bt** | **26** | **23** | **25** | **18** | **40** | **19** | **25** | **19** | **25** | **36** | **33** | **19** | **27** | **21** | **25** | **18** | **20** | **18** | **18** | **18** | **20** | **20** | **22** | **17** | **78** | **15** |  |  |
|  |  | **NBt** | **22** | **22** | **19** | **16** | **39** | **17** | **23** | **14** | **16** | **23** | **19** | **11** | **14** | **14** | **12** | **14** | **16** | **10** | **12** | **10** | **14** | **15** | **10** | **10** | **67** | **10** | **5** |  |
|  | **2** | **Bt** | **15** | **15** | **22** | **21** | **44** | **12** | **18** | **25** | **26** | **24** | **35** | **21** | **27** | **19** | **24** | **21** | **20** | **16** | **16** | **20** | **23** | **25** | **22** | **14** | **68** | **13** | **10** |  |
|  |  | **NBt** | **21** | **16** | **20** | **23** | **39** | **15** | **17** | **13** | **17** | **16** | **20** | **13** | **24** | **14** | **12** | **14** | **10** | **12** | **10** | **12** | **15** | **10** | **16** | **10** | **56** | **10** | **9** |  |
|  | **3** | **Bt** | **20** | **22** | **22** | **24** | **37** | **25** | **20** | **18** | **27** | **26** | **29** | **22** | **34** | **17** | **24** | **18** | **20** | **17** | **14** | **20** | **20** | **16** | **24** | **21** | **69** | **15** |  |  |
|  |  | **NBt** | **21** | **20** | **20** | **22** | **38** | **26** | **22** | **15** | **14** | **16** | **20** | **14** | **21** | **12** | **18** | **13** | **14** | **10** | **10** | **14** | **12** | **10** | **10** | **12** | **49** | **10** | **10** |  |
|  |  |  |  |  |  |  |  |  |  |  |  |  |  |  |  |  |  |  |  |  |  |  |  |  |  |  |  |  |  |  |
|  |  |  |  |  |  |  |  |  |  |  |  |  |  |  |  |  |  |  |  |  |  |  |  |  |  |  |  |  |  |  |
|  |  |  |  |  |  |  |  |  |  |  |  |  |  |  |  |  |  |  |  |  |  |  |  |  |  |  |  |  |  |  |
|  |  |  |  |  |  |  |  |  |  |  |  |  |  |  |  |  |  |  |  |  |  |  |  |  |  |  |  |  |  |  |
|  |  |  |  |  |  |  |  |  |  |  |  |  |  |  |  |  |  |  |  |  |  |  |  |  |  |  |  |  |  |  |
