# Supplementary material for: Biodiversity assessment and environmental risk analysis of the single line transgenic pod borer resistant cowpea
Source: PeerJ. 2024 Oct 18;12:e18094. doi: 10.7717/peerj.18094 (PMC11493023; doi:10.7717/peerj.18094)
Supplement: Supplemental Information 5 [file peerj-12-18094-s005.pdf]

Field Experience Update

Insect Live capture on the farm

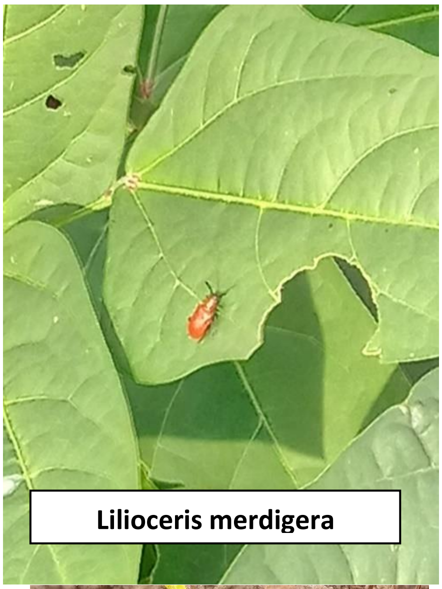

Lilioceris merdigera

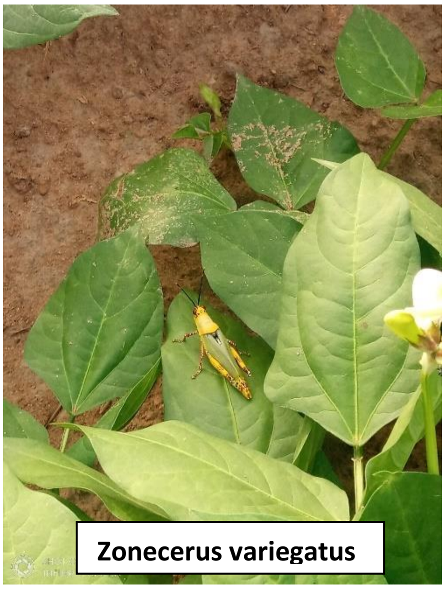

Zonecerus variegatus

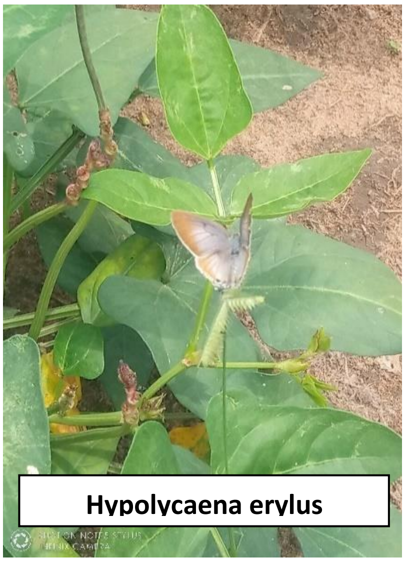

Hypolycaena erylus

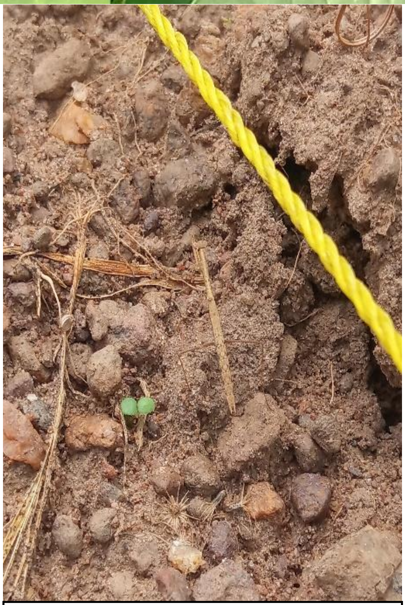

Phasmatodea spp stick insect

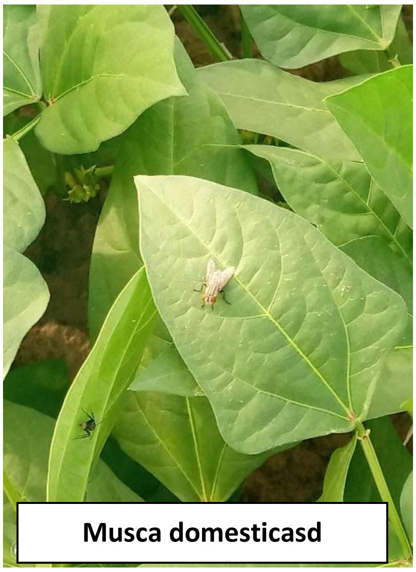

Musca domesticasd

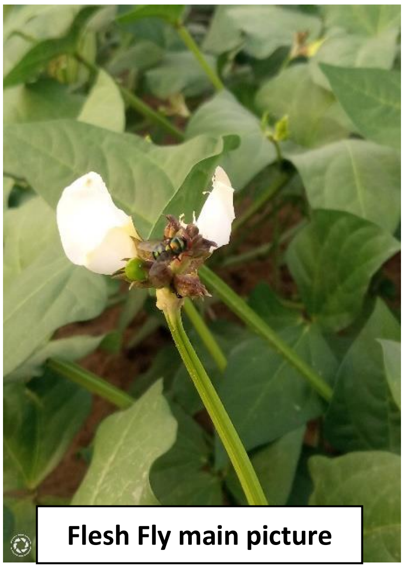

Flesh Fly main picture

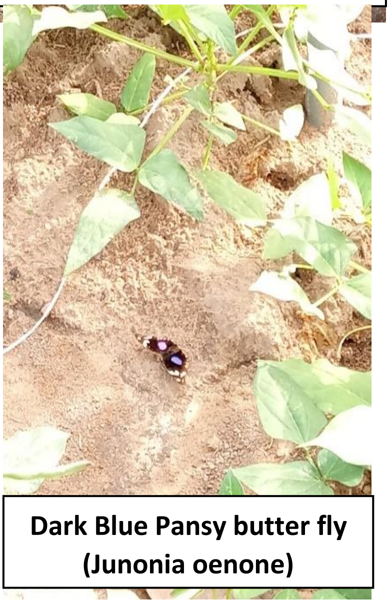

Dark Blue Pansy butter fly
(Junonia oenone)

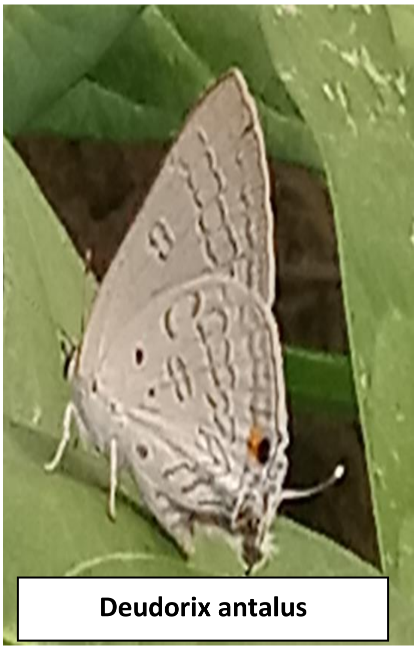

Deudorix antalus

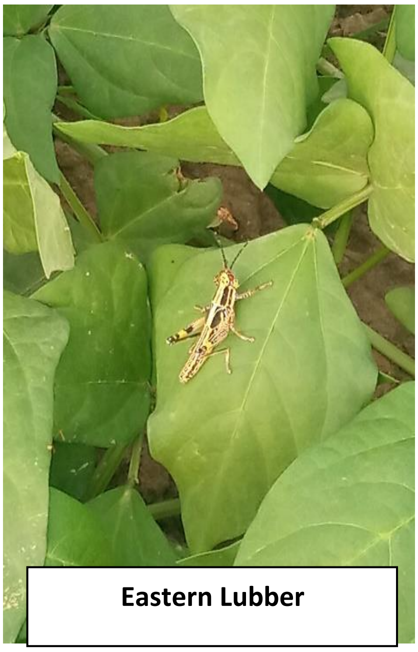

Eastern Lubber

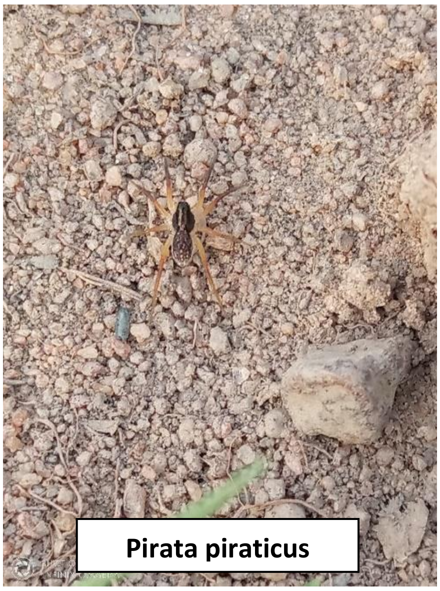
Pirata piraticus

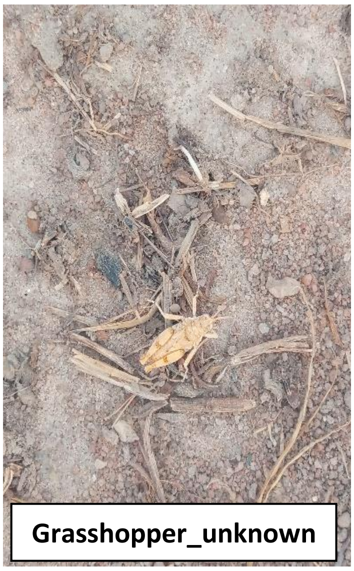
Grasshopper_unknown

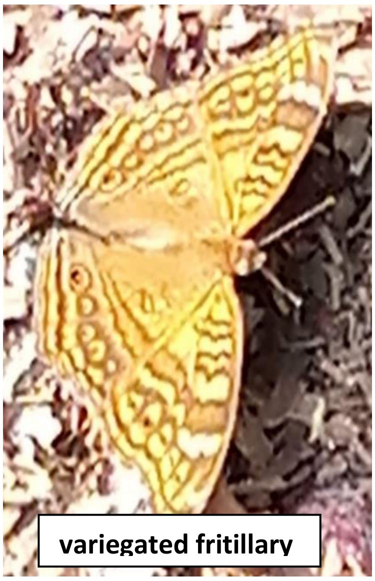
variegated fritillary

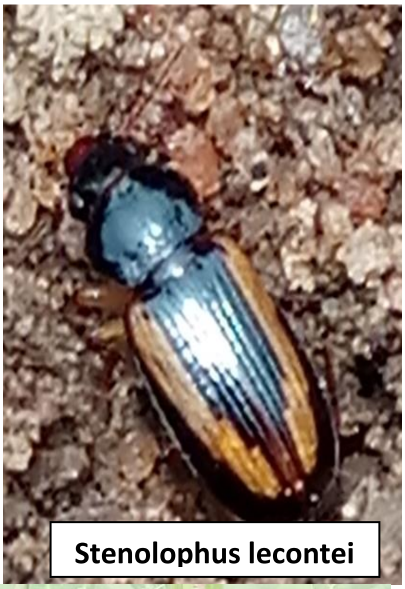
Stenolophus lecontei

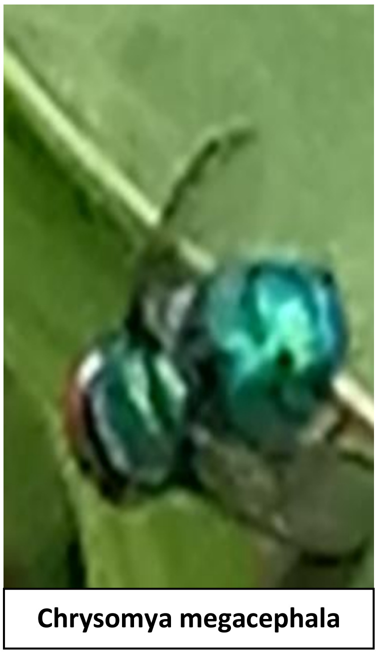
Chrysomya megacephala

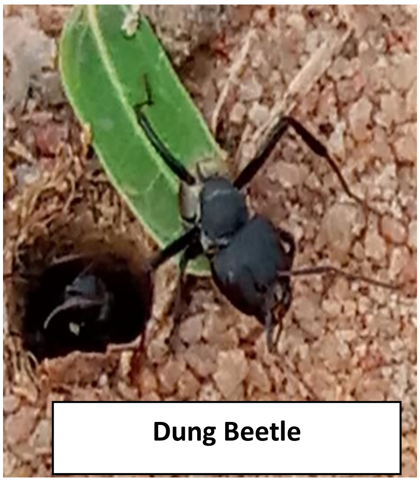
Dung Beetle

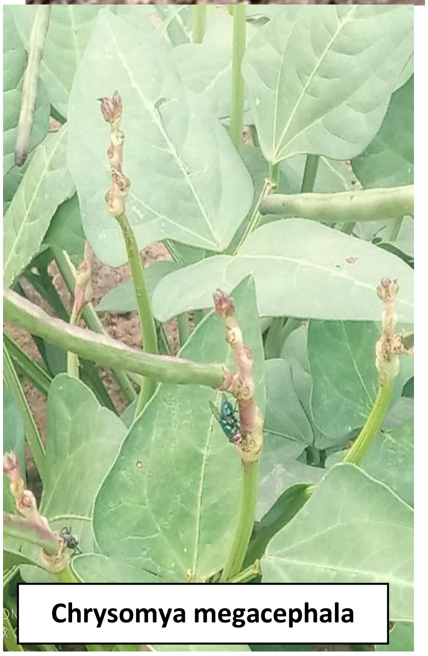
Chrysomya megacephala

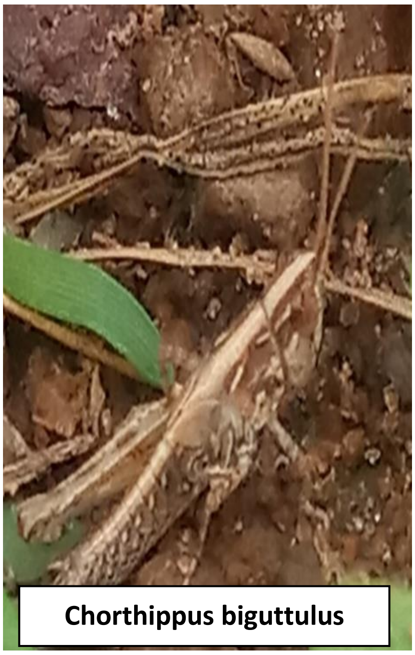
Chorthippus biguttulus

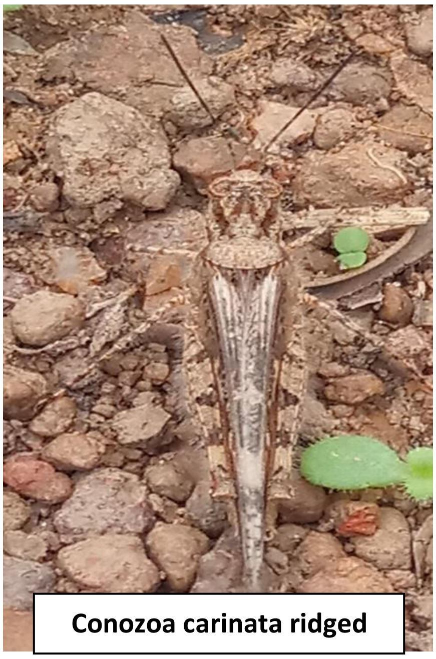
Conozoa carinata ridged

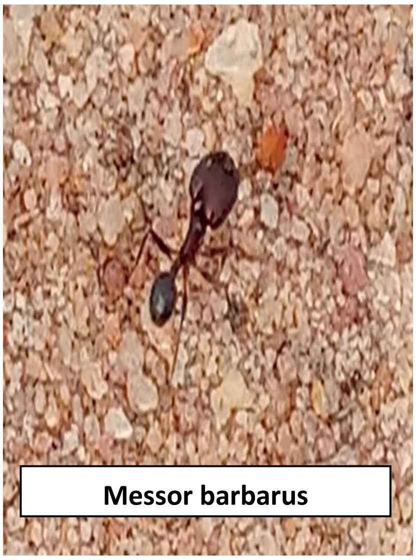
Messor barbarus

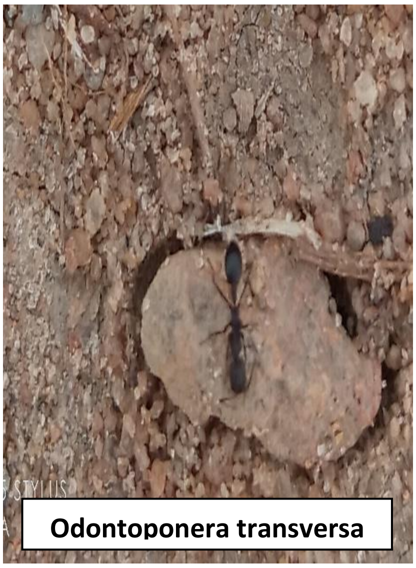
Odontoponera transversa

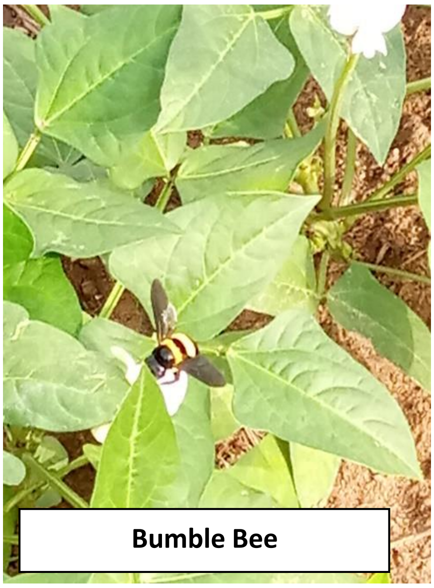
Bumble Bee

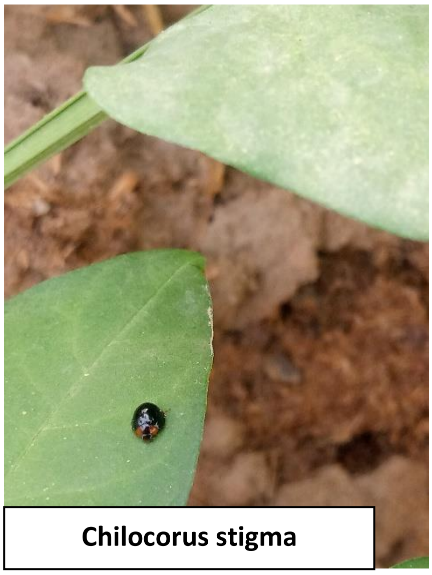
Chilocorus stigma

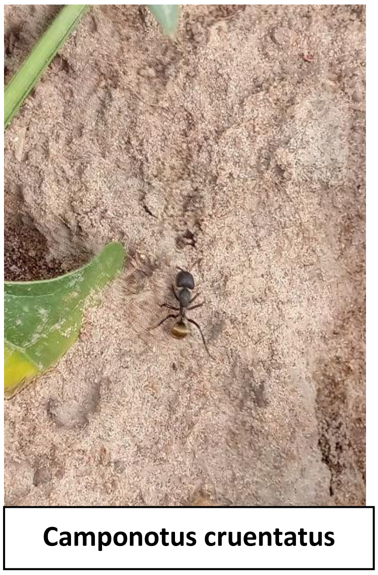
Camponotus cruentatus

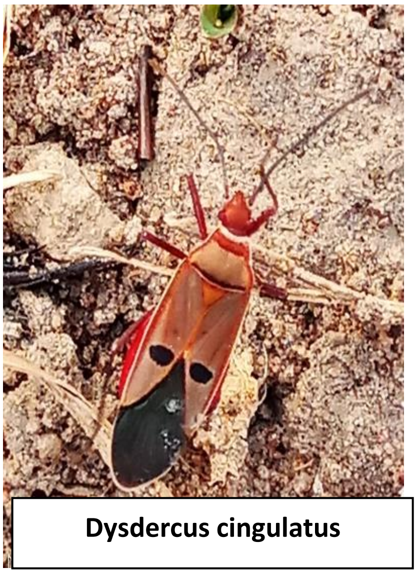
Dysdercus cingulatus

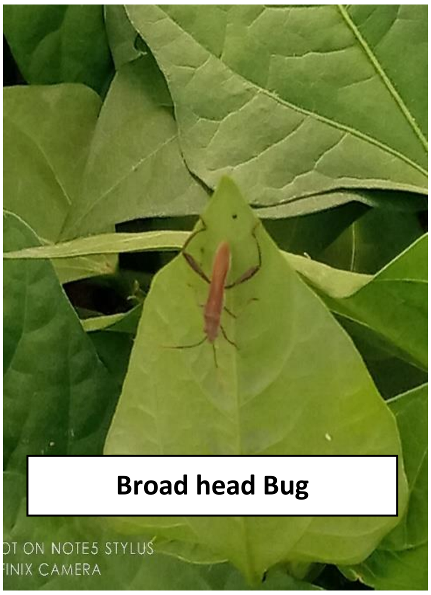
Broad head Bug

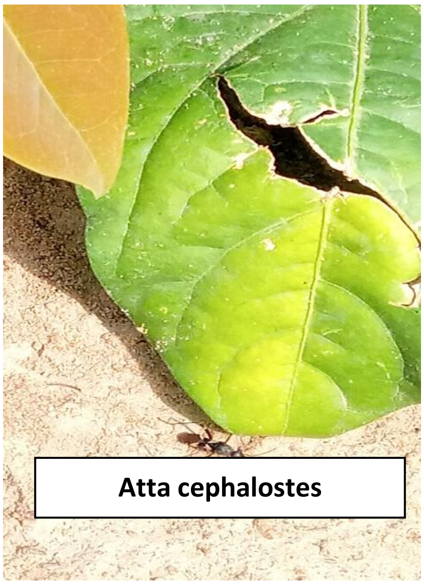
Atta cephalostes

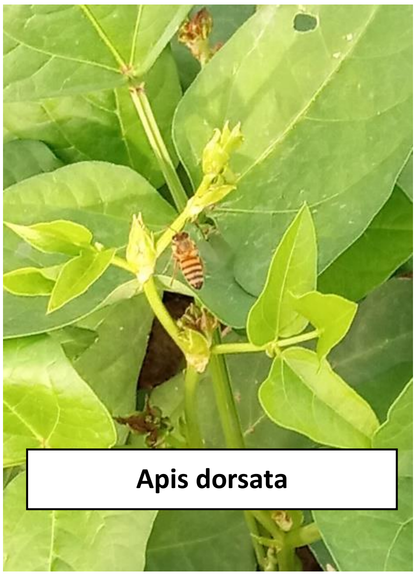
Apis dorsata

Land Preparation of the First Field

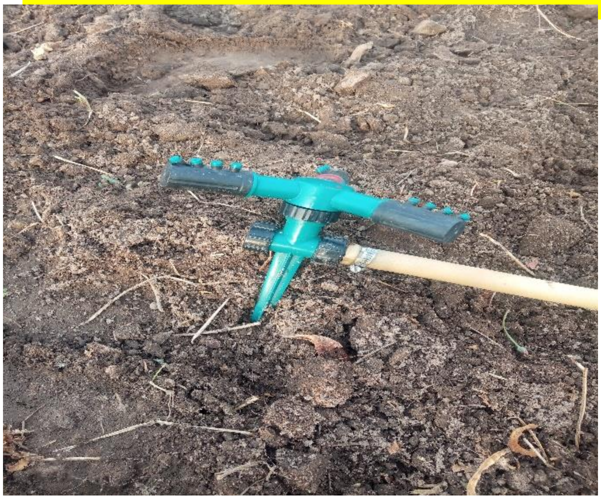

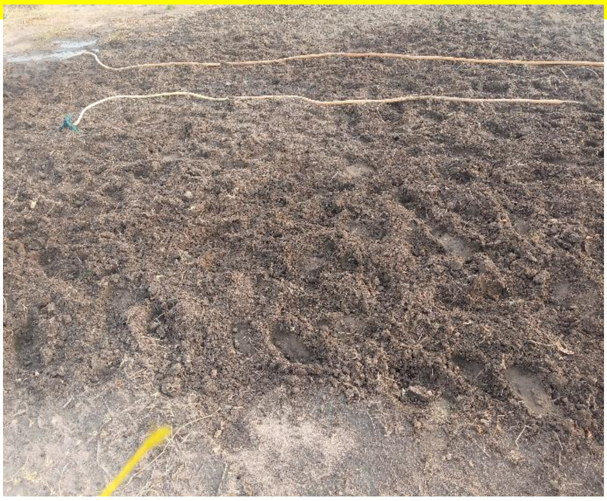

Land Preparation for Field 1_ March 4, 2022

Planting on the Field 1_ March 8, 2022

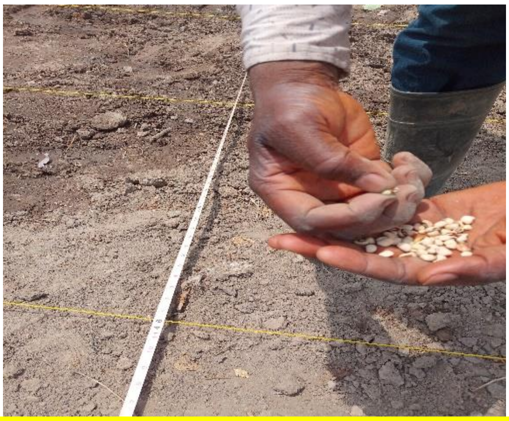

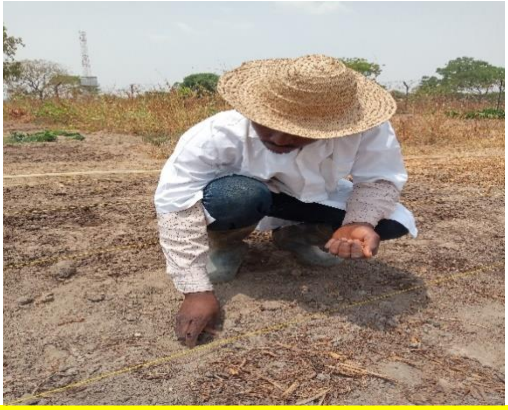

Wetting of the Crop before germination on the field

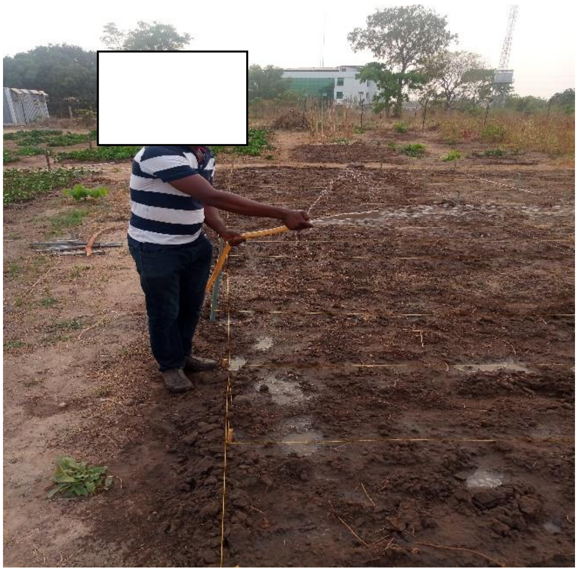

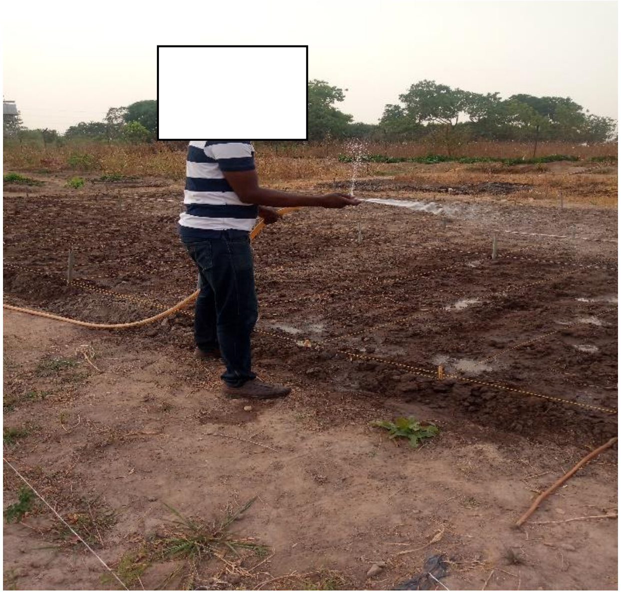

First Day of Germination for Field 1

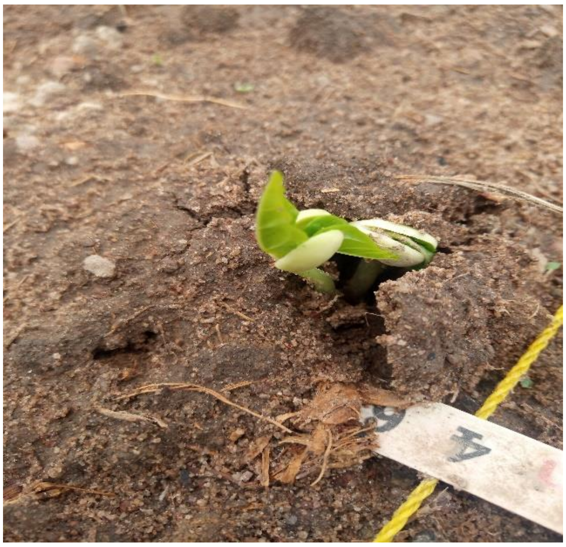

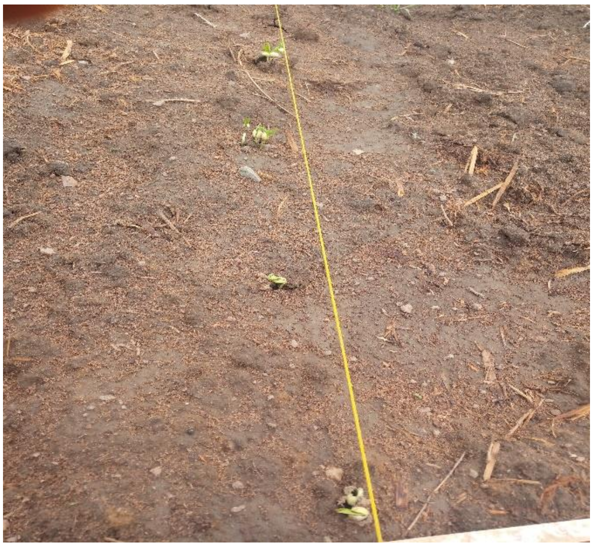

Transgenic and Non-Transgenic Cowpea Field Farm for Ecotoxicogenomic Study, 24 Days After Germination

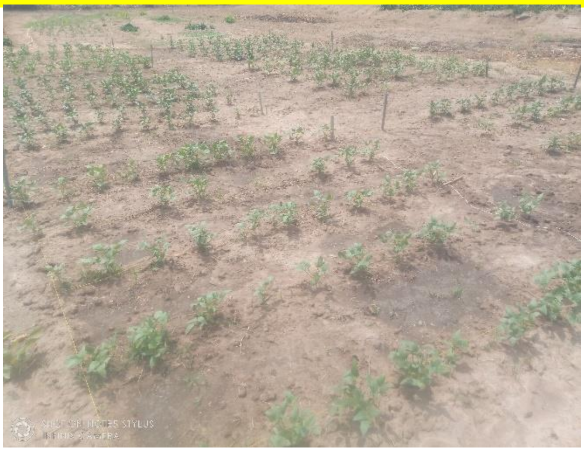

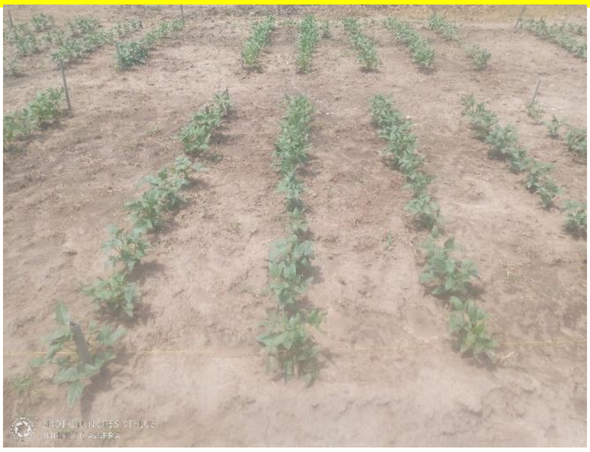

30 days after planting Pictures of Bt and NBt Cowpea

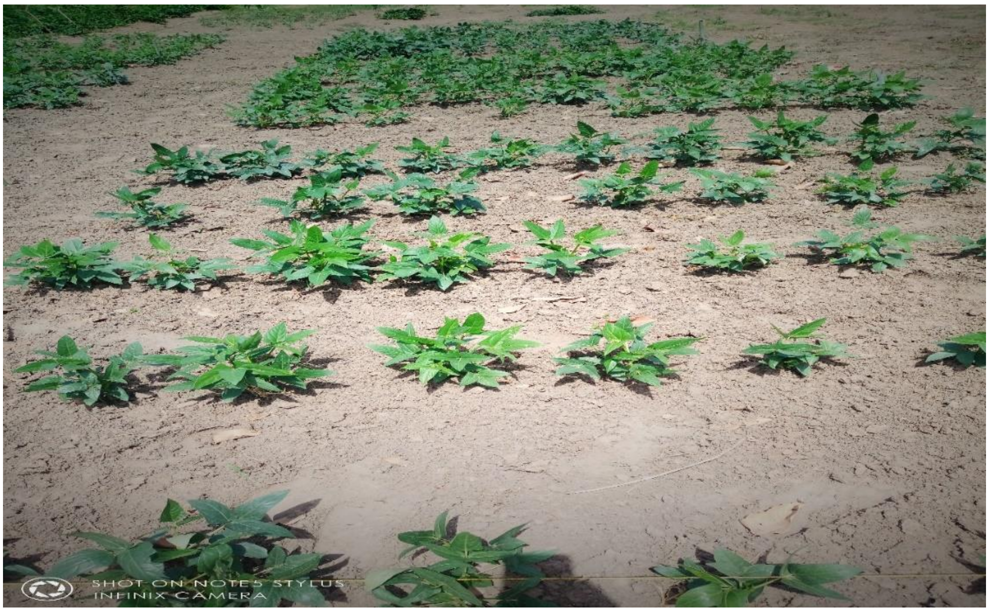

48 days after planting

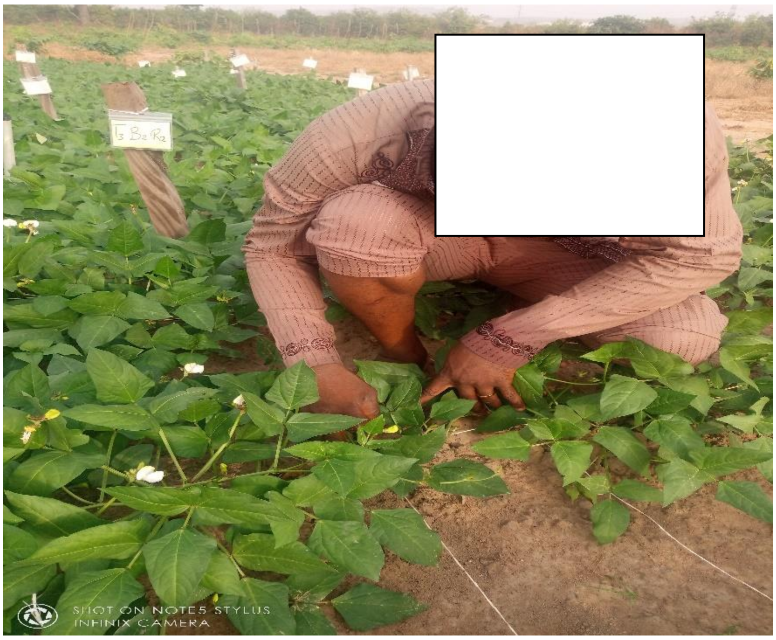

54 days after planting

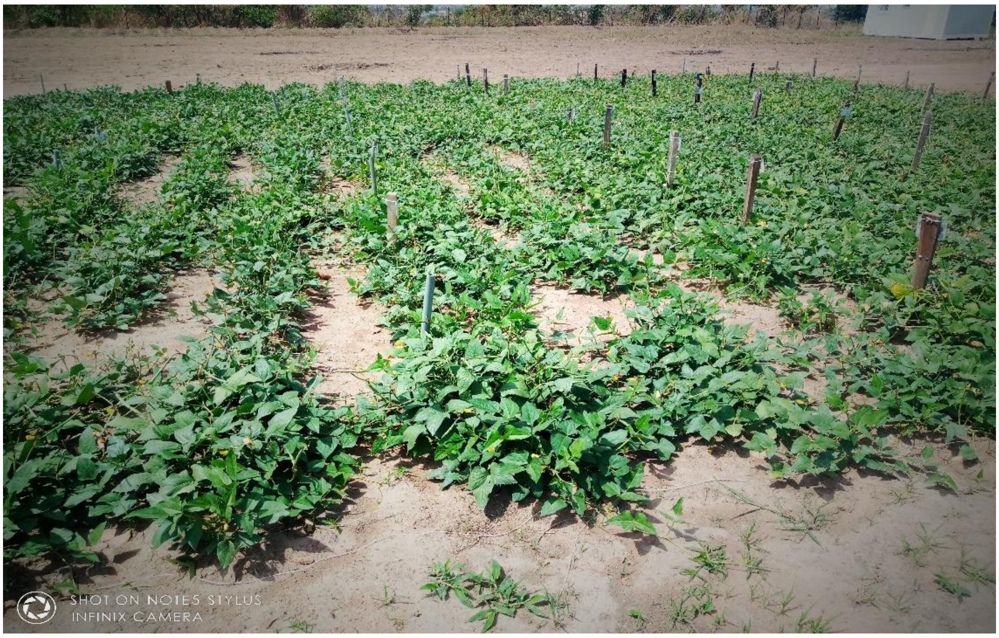

General Farm Picture

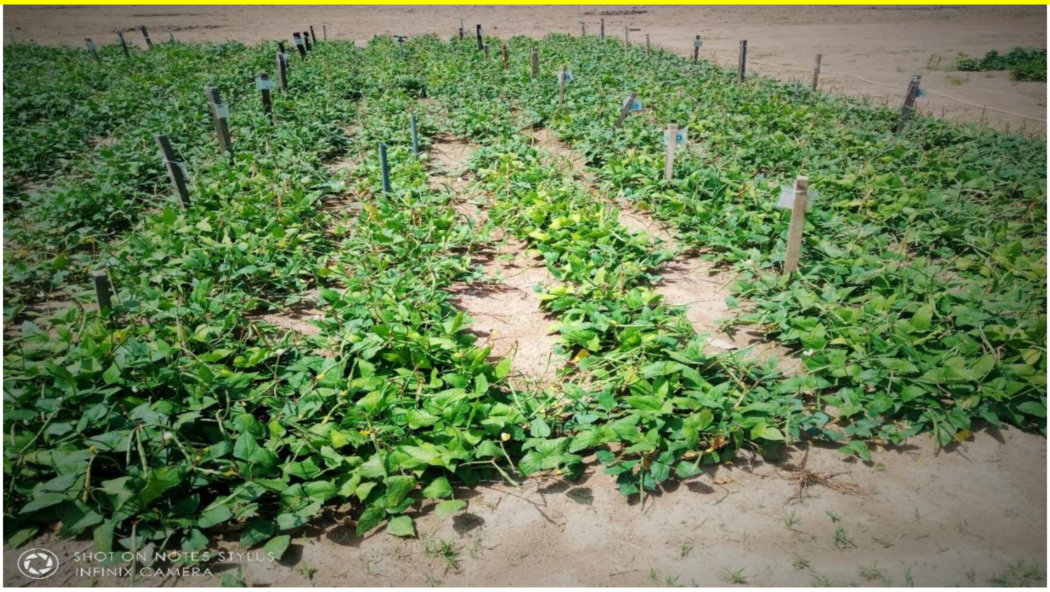

Raw Data Collection for Biodiversity study of the PBR and Non PBR Cowpea

Some Few Keynotes

i. Planting started on the 9th of March 2022 at around 1:20 pm on the CFT farms of NABDA.

ii. Germination of the seeds started around 9:11 am on the 12th March 2022. Both the *Bt* and Non *Bt* Cowpea seeds emerged the same time.

iii. Flowering (*Bt* Cowpea) started on the 15th of April 2022 but in a spontaneous and sporadic manner

iv. Flowering (N*Bt* Cowpea) started on the 1st of May 2022 but in a scattered and scanty manner (like 3 plant stand)

v. First data collection done on the 25th March 2022

Wk	Farm		Identified Insects																													
			1	2	3	4	5	6	7	8	9	10	11	12	13	14	15	16	17	18	19	20	21	22	23	24	25	26	27	28	29	30
			PP	GS	GB	CS	SaC	AE	ZV	EL	DA	MD	AC	AD	MB	SS	OT	DC	JO	BT	CM	HE	CC	SL	CB	CaM	CaC	LM	vf			
1	1	*Bt*	7	5	7	2	27	2	5	4	3	13	13	05	17	0	6	7	2	0	0	0	1	2	1	0	53	0	0			
		NBt	6	4	6	2	25	2	6	3	2	14	12	6	12	0	7	5	1	0	0	0	2	1	2	0	50	0	0			
25 Mar	2	Bt	6	6	6	3	23	1	7	5	3	15	11	07	13	1	8	3	3	0	0	0	3	2	2	0	45	0	0			
		NBt	6	5	6	4	24	3	3	5	3	12	10	5	13	0	5	2	1	0	0	0	1	2	0	0	40	0	0			
	3	Bt	5	3	4	2	24	2	5	3	2	10	09	5	11	0	7	0	1	0	0	0	1	3	1	3	23	0	0			
		NBt	3	4	5	2	22	2	4	2	1	11	10	3	12	0	6	0	2	0	0	0	1	1	1	2	25	0	0			
2	1	Bt	8	5	6	3	25	3	4	5	5	14	14	06	17	1	7	7	4	1	0	2	1	3	2	1	55	1	0			
		NBt	6	4	6	4	25	3	5	5	3	14	11	6	13	1	7	6	2	0	0	1	2	1	2	0	52	0	0			
1 Apr	2	Bt	6	6	7	5	24	2	5	7	3	14	11	7	14	0	7	3	3	01	0	1	3	2	2	0	43	1	0			
		NBt	6	3	4	3	22	3	4	5	3	13	11	6	11	1	6	2	2	0	0	01	1	3	1	0	41	1	0			
	3	Bt	6	4	5	3	25	3	6	4	1	12	10	5	12	1	7	2	2	0	0	02	2	3	2	2	25	0	0			
		NBt	5	4	5	3	24	3	4	3	2	11	11	4	12	1	5	2	2	0	0	2	1	2	2	1	23	0	0			
3	1	*Bt*	9	7	8	4	25	4	4	5	6	15	14	7	17	1	7	8	4	1	1	3	3	3	3	1	53	0	0			
		NBt	7	5	7	4	26	3	5	4	5	15	12	5	15	0	6	8	3	1	0	2	2	2	2	0	52	1	0			
8 Apr	2	*Bt*	7	6	7	5	26	3	7	7	5	13	14	7	14	0	9	4	5	0	0	2	3	3	2	1	47	1	0			
		NBt	8	4	5	4	23	2	5	6	4	12	12	7	14	1	7	5	3	1	1	2	2	3	2	1	43	1	0			
	3	Bt	7	5	6	4	25	4	7	6	3	14	11	6	15	0	7	4	3	2	1	2	2	3	3	2	24	1	0			
		NBt	6	5	4	2	22	3	4	4	2	12	11	5	10	1	6	4	4	0	0	2	2	2	2	1	21	0	0			
Flowering for *Bt* Cowpea started on the 15th of April 2022, all plants flowered the same day and in a sporadic manner: also attracted the influx of insect that facilitated pollination activities																																
4 15 Apr	1	Bt	14	10	14	7	28	6	9	8	8	19	17	10	20	3	11	11	7	3	4	5	5	4	5	2	57	3	0			
		NBt	8	6	7	5	25	3	5	5	6	13	11	6	13	1	7	6	3	1	1	2	2	1	2	0	52	1	0			
	2	Bt	13	11	12	8	29	5	11	8	9	17	19	9	17	5	11	6	5	4	6	6	5	6	7	3	51	3	0			
		NBt	8	5	7	5	22	2	6	6	5	13	12	6	12	2	8	3	2	3	3	3	2	3	4	1	45	1	0			
	3	Bt	15	7	9	7	28	6	9	9	5	18	15	8	17	2	11	9	7	3	2	3	4	6	5	4	58	3	0			
		NBt	8	6	5	4	23	4	6	5	3	13	12	6	11	1	7	5	3	1	1	1	2	4	2	2	22	1	0			
5	1	**Bt**	17	13	17	9	30	9	11	12	14	21	19	12	23	5	14	13	10	5	5	6	7	7	8	4	63	5	3			

22 Apr		NBt	9	5	3	4	7	5	5	7	4	13	11	5	14	2	7	5	6	1	2	3	2	3	2	2	50	1	2			
	2	Bt	15	13	15	10	32	9	13	11	12	19	22	11	22	7	14	8	7	6	8	7	7	7	9	4	56	5	4			
		NBt	6	5	4	3	21	2	5	4	3	07	11	6	13	1	7	6	4	2	1	2	1	1	2	2	39	1	1			
	3	Bt	16	12	13	11	32	9	10	12	7	20	17	11	21	4	14	14	13	7	6	9	8	8	10	7	60	7	2			
		NBt	7	9	6	4	25	3	5	7	4	11	13	7	13	0	8	6	5	2	1	2	3	2	3	4	22	1	3			
6 29 Apr	1	Bt	18	15	17	10	29	10	10	13	15	23	20	14	24	7	13	12	12	7	6	8	9	10	9	7	67	7	5			
		NBt	11	7	5	7	11	5	6	7	7	16	15	9	14	3	7	8	7	3	4	3	4	4	5	3	56	3	3			
	2	Bt	16	13	17	13	33	10	12	15	14	24	24	12	22	9	15	10	9	7	10	11	9	8	10	7	57	7	4			
		NBt	9	6	5	5	23	3	7	6	5	9	14	5	11	3	5	6	4	3	4	3	3	4	3	3	41	2	2			
	3	Bt	18	13	17	13	33	11	12	12	8	22	18	13	23	6	16	15	14	9	8	13	9	9	13	11	62	8	4			
		NB*t*	8	10	7	5	27	5	4	6	6	15	11	8	15	2	9	7	4	3	2	3	4	4	5	2	27	3	2			
Flowering for N*Bt* started on the 1st May in a disordered and scanty manner with three plant stand																															
7 06 May	1	*Bt*	22	17	19	12	33	11	13	14	17	24	24	15	26	8	14	15	14	10	7	9	11	11	10	9	69	8	5			
		N*Bt*	15	9	6	9	13	7	9	11	8	18	16	8	15	5	8	10	9	5	6	5	5	3	7	4	55	5	4			
	2	*Bt*	17	15	16	14	35	13	13	16	17	25	26	15	23	11	17	12	10	10	12	13	12	9	12	9	59	9	5			
		N*Bt*	9	7	8	7	28	6	8	8	7	10	15	7	13	5	7	7	5	5	6	5	6	5	4	6	46	4	5			
	3	*Bt*	20	15	20	16	34	16	15	12	9	25	19	15	24	7	17	17	17	12	10	15	11	12	15	11	65	10	6			
		N*Bt*	10	12	9	8	29	7	5	7	9	17	12	9	17	3	10	8	6	5	4	4	6	5	6	4	34	5	3			
8 13 May	1	*Bt*	22	18	19	11	35	13	13	13	19	23	26	15	26	10	15	17	15	12	9	10	12	12	9	10	72	10	4			
		N*Bt*	12	10	8	7	14	9	10	12	9	19	15	10	15	6	10	11	9	7	4	6	7	5	6	5	60	7	5			
	2	*Bt*	15	17	17	15	33	15	15	19	18	22	27	14	25	10	16	14	11	11	10	14	15	11	11	12	54	7	6			
		NBt	7	8	10	8	25	7	10	9	10	11	13	9	14	7	6	7	7	7	8	6	7	8	5	8	48	5	3			
	3	Bt	22	17	18	18	37	19	16	14	20	26	22	16	25	8	20	18	18	13	12	16	13	12	17	13	67	11	5			
		NBt	11	13	10	10	28	10	8	9	10	16	13	11	18	5	12	9	9	7	7	6	8	4	7	7	36	4	2			
9 20 May	1	*Bt*	25	20	23	12	37	15	17	14	20	25	28	18	27	13	16	17	16	14	11	12	14	13	13	11	73	11	6			
		N*Bt*	14	11	10	7	13	11	12	11	9	20	16	11	14	8	9	11	10	8	5	8	8	7	7	7	59	7	3			
	2	*Bt*	14	19	19	16	36	17	14	19	19	22	30	15	25	12	18	14	13	11	13	17	18	14	13	13	56	8	6			
		NBt	9	9	12	10	27	10	11	10	11	12	14	10	16	9	9	7	7	9	9	7	9	9	6	7	49	6	4			
	3	Bt	25	19	19	21	35	22	17	13	22	27	26	16	28	9	21	18	18	15	12	17	15	12	18	14	63	9	7			
		NBt	10	12	11	11	27	11	9	10	9	15	16	11	19	6	11	12	10	9	8	9	9	6	8	9	34	6	5			
10 27 May	1	Bt	26	19	24	14	39	16	19	15	22	27	29	17	28	15	19	16	17	15	13	13	15	16	15	12	74	13	4			
		NBt	15	13	11	6	12	12	13	09	12	20	17	10	15	9	10	10	12	9	7	8	9	9	8	6	62	6	3			
	2	Bt	15	18	21	17	37	16	15	22	20	21	33	16	26	14	19	15	14	13	12	17	18	15	18	14	62	9	5			
		NBt	10	9	14	11	25	11	12	11	10	12	15	09	17	9	10	9	10	8	10	8	10	10	9	8	52	7	6			
	3	Bt	23	19	18	22	36	23	18	15	24	26	27	19	29	13	23	17	17	16	14	18	16	13	19	16	66	11	6			
		NBt	09	10	12	13	26	13	10	12	11	17	15	12	18	9	13	13	9	6	9	11	10	7	9	11	39	4	3			

11	1	Bt	27	21	25	16	37	17	22	16	21	29	33	15	25	17	21	17	17	15	14	17	16	17	18	13	77	14	7			
		NBt	16	15	12	17	14	14	17	11	13	19	18	10	15	11	9	12	13	10	8	7	11	10	7	6	65	8	5			
03 June	2	Bt	16	17	23	18	39	16	14	23	24	22	32	19	27	16	21	17	15	14	12	17	20	22	19	12	65	11	5			
		NBt	11	9	15	14	23	10	8	12	13	15	17	11	21	10	11	10	8	9	07	9	13	12	11	9	51	8	3			
	3	Bt	26	20	21	23	37	24	20	16	26	25	28	21	33	15	22	16	19	15	12	19	18	15	22	20	67	13	7			
		NBt	11	14	14	16	23	14	13	09	10	16	17	13	18	10	14	12	12	8	7	14	12	6	9	12	42	7	6			
12	1	Bt	25	22	24	17	39	18	24	17	23	35	32	17	26	19	24	17	18	16	16	17	18	18	21	15	77	13	6			
		NBt	12	15	09	10	16	13	15	12	14	22	17	09	13	12	10	13	15	09	10	8	12	13	8	7	66	8	3			
10 June	2	Bt	22	18	23	20	42	16	16	24	25	23	34	20	26	17	23	20	18	15	14	18	22	24	21	13	67	11	7			
		NBt	13	11	16	13	27	12	9	11	15	15	19	11	22	12	11	12	9	10	8	10	14	11	14	9	55	6	4			
	3	Bt	24	21	21	23	37	24	20	16	26	25	28	21	33	15	22	16	19	15	12	19	18	15	22	20	67	13	5			
		NBt	11	12	15	17	24	16	12	14	12	14	18	12	20	11	16	11	12	9	5	12	10	7	9	10	47	6	6			

Keys

1. ***Pirata piraticus (PP) (Spider)***
2. **Grasshopper spp (GS) (now identified as *Conozoa hylena)***
3. ***Graphoderus bilineatus (GB) (Beetle)***
4. ***Chilocorus stigma (CS) (Lady bug)***
5. **Sarcophaga crassipalpis Macquart (SaC) (Flesh fly)**
6. ***Alydus eurinus (AE) (Broad-headed Bug)***
7. ***Zonecerus variegatus (ZV) (Grasshopper)***
8. **Eastern Lubber (EL) (Grasshopper)**
9. ***Deudorix antalus (DA) (Butterfly)***
10. **Musca domestica (MD) (housefly)**
11. **Atta cephalostes (AC) (Leaf Cutting Ant)**
12. ***Apis dorsata (AD) (Giant Honeybee)***
13. ***Messor barbarous (MB) (Harvester Ant)***
14. ***Scarabaeus satyrus (sacred Dung beetle)*** *– Worshipped by the ancient Egyptians and believed to be sacred because it has no female but yet reproduce…* ***read more about it***
15. ***Odontoponera transversa (OT) (Ant)***

16. ***Dysdercus cingulatus (Cotton bug) –*** *peculiar only to farm 2 because it is close to the cotton farm. It means that pbr cowpea is safe to any environment where its s planted and since insects peculiar such environment will be able to easily adapt without any resistance. If pbr cowpea is planted in regions where for example, thermites are common, inhabited insects which is in this scenario is the thermite will be able to adapt with it still successfully.*
17. **Junonia oenone (JO) (butterfly)**
18. **Bombus terrestris (BT) (Bomble Bee)**
19. **Chrysomya megacephala (CM) (Oriental Laterine Fly)**
20. **Hypolycaena erylus (HE) (butterfly)**
21. ***Conozoa carinata (CC) (*ridged grasshopper*)***
22. **Stenolophus lecontei (SL) (beetle)**
23. **Chorthippus biguttulus (CB) (grasshopper)**
24. **Carausius morosus (CaM) (Phasmatodea stick insect)**
25. **Camponotus cruentatus (CaC) (Ant)**
26. **Lilioceris merdigera (LM) (Beetle) –** also called red lilybeetle, scarlet lily beetle, lily leaf beetle is a leaf beetle that eats the leaves, stem, buds, and flowers of lilies, fritillaries and other members of the Liliaceae family
27. **Variegated fritillary (vf) (butterfly)…** observed towards the last three weeks and more frequent after rain fall

Insect identification, Field trials farms for the Biodiversity study of Transgenic and non-Transgenic Cowpea

A Weekly Insect Count Duration Between March and April 2022			
SN	*Insect identification*	*Conventional Cowpea Field Average Insect count*	*GM Cowpea Field Average Insect Count*
1	*Pirata piraticus Spider* 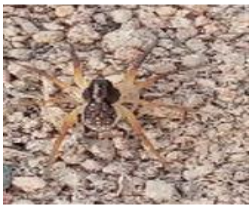	*17*	*13*
2	Grasshopper spp now identified as *Conozoa hyalina* 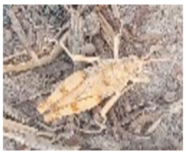	*23*	*17*
3	*Graphoderus bilineatus (Beetle)* 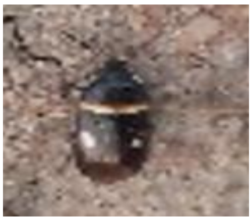	*9*	*11*
4	*Chilocorus stigma* 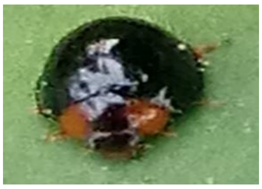	*11*	*12*
5	Sarcophaga crassipalpis Macquart (SaC) Flesh Fly 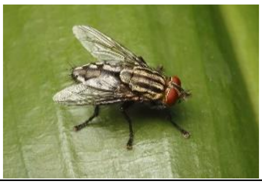	*58*	*67*
6	*Alydus eurinus* 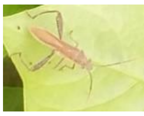	*23*	*25*
7	*Zonecerus variegatus grasshopper* 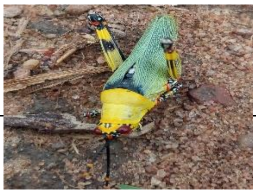	*22*	*23*

8	**Eastern Lubber grasshopper** 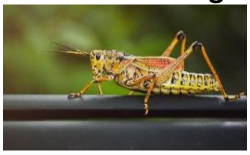	***27***	***25***
9	***Deudorix antalus butterfly*** 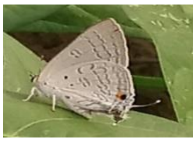	***32***	***28***
10	**Musca domestica housefly** 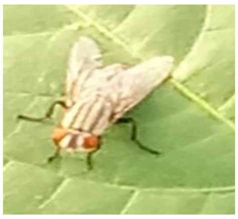	***54***	***61***
11	**Atta cephalotes (leaf cutter ant)** 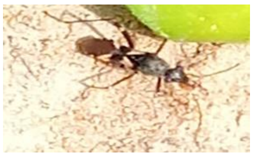	***131***	***127***
12	***Apis dorsata (honeybee)*** 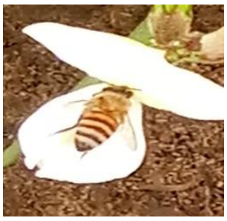	***31***	***33***
13	***Messor barbarous (ants)*** 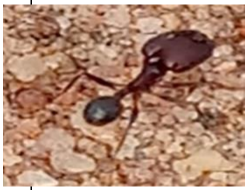	***53***	***57***
14	***Scarabaeus satyrus (sacred Dung beetle)*** *Worshipped by ancient egyptians and believed to be sacred…browse about it* 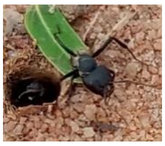	***12***	***13***
15	***Odontoponera transversa (Ant)*** 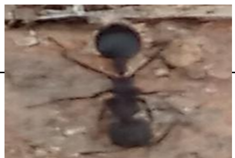	***68***	***71***

16	*Dysdercus cingulatus (Cotton bug)*<br>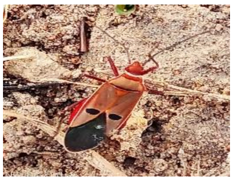<br>*Common to only farm 2 & 1 (most likely because of the cotton farm close to farm 2 because it is a cotton insect)*	*17*	*21*
17	**Junonia oenone butterfly**<br>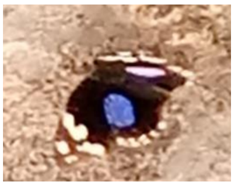	*29*	*23*
18	**Bombus terrestris (BT) (Bumble Bee)**<br>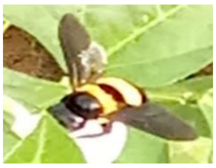<br>*Common to only famr 1 and 2*	*13*	*11*
	**Second Phase Identification taken 2nd May 2022**		
19	**Chrysomya megacephala (oriental latrine fly)**<br>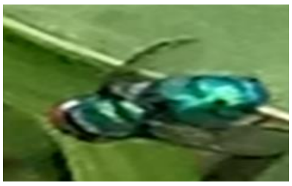<br>Common to only farm 1 and 2	*34*	*32*
20	**Hypolycaena erylus butterfly**<br>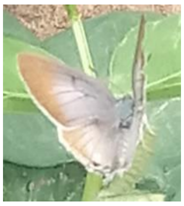	*47*	*49*
21	***Conozoa carinata* ridged grasshopper**<br>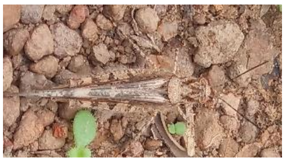	*23*	*21*

22	**Stenolophus lecontei beetle**<br>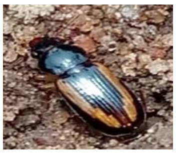<br>**Common to only farm 3**	*17*	*13*
23	**Chorthippus biguttulus grasshopper**<br>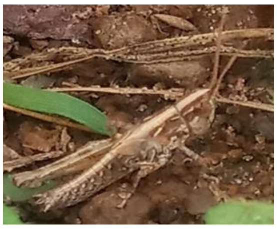	*19*	*20*
24	**Carausius morosus (Phasmatodea stick insect**<br>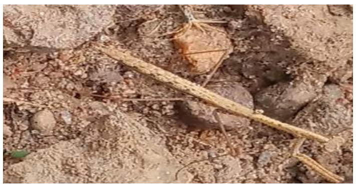<br>**Common to only farm three**	*11*	*10*
25	**Camponotus cruentatus (Ant)**<br>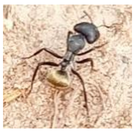	*105*	*102*
26	**Lilioceris merdigera (red lilybeetle)**<br>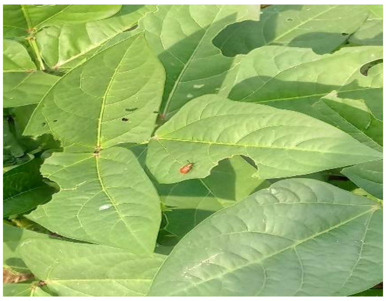<br>*Caught in the act, red lily beetle otherwise known as leaf eater caught feeding on non-transgenic cowpea on the farm.*	*21*	*19*
27	**variegated fritillary (butterfly)**<br>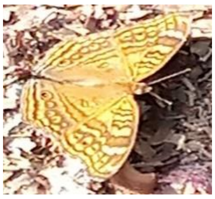		

Collected Insects Classification/ Categorization

SN	Herbivores	Predators	Parasitoids	Carnivore	Omnivore
			Graphoderus bilineatus (Beetle)		Musca domestica
	Alydus eurinus	*Chilocorus stigma*		Sarcophaga crassipalpis Macquart (SaC) Flesh Fly	
	Zonecerus variegatus grasshopper	Camponotus cruentatus	*Scarabaeus satyrus (sacred Dung beetle)*		
	Eastern Lubber grasshopper	*Pirata piraticus*	Lilioceris merdigera (red lilybeetle)		
	Deudorix antalus butterfly		Stenolophus lecontei beetle		
	Atta cephalotes ants		Chrysomya megacephala		
	Apis dorsata				
	Messor barbarus				
	Odontoponera transversa (Ant)				
	Dysdercus cingulatus				
	Junonia oenone				
	Bombus terrestris				
	Hypolycaena erylus				

	Conozoa carinata				
	**Chorthippus biguttulus**				
	**Carausius morosus**				
	**variegated fritillary**				
	**Grasshopper spp**				
